# Cik1 and Vik1 accessory proteins confer distinct functions to the kinesin-14 Kar3

**DOI:** 10.1242/jcs.260621

**Published:** 2023-06-13

**Authors:** Zane J. Bergman, Jonathan J. Wong, David G. Drubin, Georjana Barnes

**Affiliations:** Department of Molecular and Cell Biology, University of California, Berkeley, CA 94720, USA

**Keywords:** Kinesin-14, Reconstitution, Microtubules, Kar3, Cell cycle

## Abstract

The budding yeast *Saccharomyces cerevisiae* has a closed mitosis in which the mitotic spindle and the cytoplasmic microtubules (MTs), both of which generate forces to faithfully segregate chromosomes, remain separated by the nuclear envelope throughout the cell cycle. Kar3, the yeast kinesin-14, has distinct functions on MTs in each compartment. Here, we show that two proteins, Cik1 and Vik1, which form heterodimers with Kar3, regulate its localization and function within the cell, and along MTs in a cell cycle-dependent manner. Using a yeast MT dynamics reconstitution assay in lysates from cell cycle-synchronized cells, we found that Kar3-Vik1 induces MT catastrophes in S phase and metaphase, and limits MT polymerization in G1 and anaphase. In contrast, Kar3-Cik1 promotes catastrophes and pauses in G1, while increasing catastrophes in metaphase and anaphase. Adapting this assay to track MT motor protein motility, we observed that Cik1 is necessary for Kar3 to track MT plus-ends in S phase and metaphase but, surprisingly, not during anaphase. These experiments demonstrate how the binding partners of Kar3 modulate its diverse functions both spatially and temporally.

## Teaser

**Summary:** We show, through biochemical reconstitution experiments and live-cell imaging, that functions and localization of Kar3 are dictated by the stage of the cell cycle and the activities of two Kar3 accessory binding partners – Cik1 and Vik1.

## INTRODUCTION

Microtubules are highly dynamic polymers with a myriad of functions in cells, including providing mechanical support for large cellular structures, acting as tracks for intracellular cargo and forming the mitotic spindle to faithfully segregate chromosomes to daughter cells (Chaaban and Brouhard, 2017). MTs assemble into a variety of structures and perform diverse functions due to interactions with a host of microtubule-associated proteins (MAPs) that control MT interactions and assembly dynamics. MAPs can affect MT assembly and disassembly through various mechanisms, including as polymerases, depolymerases, stabilizers and severing factors (Bowne-Anderson et al., 2015; Roll-Mecak and McNally, 2010). One particularly important class of MAPs is the motor proteins, which generate forces on MTs for moving cargos within the cell, separating spindle poles and chromosomes, and bending cilia and flagella (Hirokawa et al., 2009; Lindemann and Lesich, 2010; Walczak et al., 1998). The concerted effects of these many different types of MAP ultimately dictate MT form and function. In the budding yeast *Saccharomyces cerevisiae*, MTs in vegetatively growing cells have only one essential function: to faithfully segregate sister chromatids to daughter cells during mitosis (Huffaker et al., 1988). The various steps of this complex process – which include spindle assembly and movement to the bud neck, and chromosome segregation − occur concurrently in budding yeast due to, in large part, the distinct properties independently imparted by MAPs in the nucleus and cytoplasm because yeast cells have a closed mitosis. The MAPs responsible for differential regulation of MTs can be completely excluded from one compartment, as in the case of Kip2 and dynein, which exclusively reside in the cytoplasm, and Kip1, Cin8 and Ase1, which are exclusively found in the nucleus (Hildebrandt and Hoyt, 2000; She et al., 2019). Alternatively, some MAPs might be post-translationally modified differentially across compartments to give rise to these different functions in the nucleus and in the cytoplasm (van der Vaart et al., 2017).

In the case of Kar3, previous studies have established that its subcellular localization is determined by its binding partners Cik1 and Vik1. In the nucleus, the heterodimer Kar3-Cik1 (hereafter referred to as Kar3Cik1) generates spindle forces (Hoyt et al., 1993; Saunders et al., 1997a), properly arrays interpolar MTs (Molodtsov et al., 2016), has a role in kinetochore capture (Liu et al., 2011; Middleton and Carbon, 1994) and transports newly captured chromosomes along MTs (Tanaka et al., 2005, 2007). The Kar3-Vik1 (hereafter referred to as Kar3Vik1) heterodimer is only cytoplasmic (Manning et al., 1999) and functions of this complex are presently not known. Previous work has shown that absence of Kar3 increases number and length of cytoplasmic MTs (Huyett et al., 1998; Saunders et al., 1997b), suggesting a role in assembly regulation and providing evidence that Kar3Vik1 focuses MT minus-ends on the cytoplasmic face of the spindle pole body (SPB), the MT organizing center in budding yeast (King et al., 2021). Roles for the two different Kar3 heterodimers in the regulation of MT dynamics through the cell cycle have not yet been elucidated.

Dissecting the functions specific to each type of Kar3 heterodimer provided a unique opportunity to investigate a novel mode of kinesin regulation; however, this has been challenging. The activity of Kar3 heterodimers on MTs was first described as a minus-end-directed translocation with low processivity (Endow et al., 1994), a property common to other kinesin-14 family members. Subsequent Kar3 studies provided evidence for its potential to switch between plus-end- and minus-end-directed movement (Molodtsov et al., 2016), or to stably associate with a growing plus-end (Sproul et al., 2005), possibly when in complex with Bim1, the budding yeast homolog of MAPRE family members (also known as EB1) (Kornakov et al., 2020; Ogren et al., 2022; Sproul et al., 2005). In addition, although early work with Kar3 provided evidence for plus-end depolymerase activity (Sproul et al., 2005), later work failed to document this activity (Mieck et al., 2015).

The *in vitro* assays of Kar3 function and regulation cited above did provide insights into possible Kar3 functions, but inconsistency of the results is also a source of confusion. While these studies are pioneering efforts to elucidate Kar3 function, they might have suffered from complications, such as use of heterogeneous assay constituents and innovative but, nevertheless, non-physiological recombinant fusion proteins to stably construct the two Kar3 heterodimers. These earlier reconstitution assays might also have suffered from a lack of post-translational modifications present in native yeast cells. Importantly, these modifications are likely to be specific to the cell cycle phase. Also missing from previous *in vitro* MT dynamics studies using purified proteins is the complete panoply of microtubule-associated proteins (MAPs) present within the cell and, thus, the possibility to uncover emergent properties of this system in its full complexity.

We have previously developed a biochemical reconstitution assay that uses yeast lysates and harnesses the genetic tractability of budding yeast while enabling imaging of assembly dynamics of single MTs, which is very difficult in living cells (Bergman et al., 2019). This assay uses total internal reflection fluorescence (TIRF) microscopy and the lysates are prepared from cells arrested at specific cell cycle stages. Here, we utilize this *in vitro* assay to reveal effects of two distinct Kar3 heterodimers on MT dynamics in the context of the complexity of a cell at each stage of the cell cycle. We also describe association of Kar3 heterodimers with, and locomotion along, MTs. In total, we show that Kar3Cik1 and Kar3Vik1 have similar but distinct motility profiles and functions that are regulated in the cell cycle and intrinsic to the respective heterodimer. Complementary *in vivo* studies demonstrate the applicability of these results to live cells.

## RESULTS

### Kar3 heterodimer localization is cell cycle dependent

**Fig. 1.**
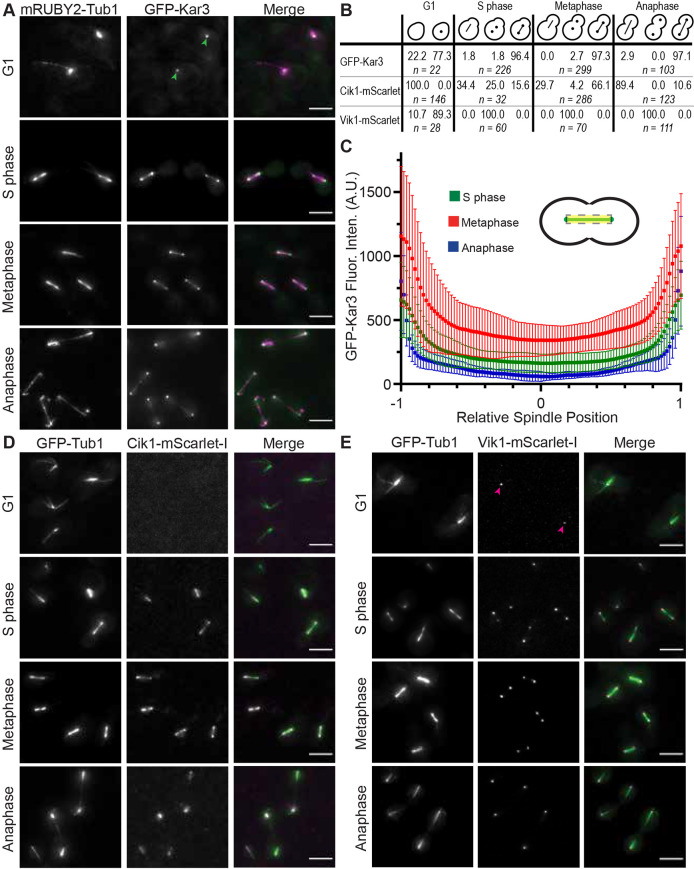
**Cell cycle-dependent localization of Kar3 heterodimers.** (A) Localization of GFP-Kar3 (green) with mRUBY2-Tub1 (magenta) during G1 phase, S phase, metaphase or anaphase. Arrowheads indicate GFP-Kar3 near SPBs. (B) Percentage of cells with localization patterns of GFP-Kar3, Cik1-mScarlet or Vik1-mScarlet at each stage of the cell cycle. *n*=number of cells counted. (C) Fluorescence intensity of GFP-Kar3 across spindles in metaphase-arrested cells. Plotted background-subtracted fluorescence intensity across spindles from the center of one pole to that of the other was divided into 100 parts, with the midzone as 0. *n*=50. Error bars indicate the mean±s.d. A.U., arbitrary units. (D) Cik1-mScarlet-I (magenta) localization along GFP-Tub1 (green) MTs throughout the cell cycle. (E) Vik1-mScarlet-I (magenta) localization along GFP-Tub1 (green) MTs throughout the cell cycle. Arrowheads indicate Vik1-mScarlet-I near SPBs. Scale bars: 5 µm.

Because the subcellular localization of proteins provides important information about their biological function, we set out to fully describe the localization of the two types of Kar3 heterodimer through the cell cycle. We generated *S*. *cerevisiae* strains that express GFP-Kar3 fusion protein from the native *KAR3* locus. These strains also express *mRUBY2-TUB1* (Markus et al., 2015) used for visualizing MTs, and temperature-sensitive *cdc* alleles that allow arrest at different cell cycle stages. The strains were arrested at G1 (*cdc28-4*), S phase (*cdc7-1*), metaphase (*cdc23-1*) or late anaphase (*cdc15-2*) (Goranov et al., 2009). Arrested cells were observed by time-lapse wide-field fluorescence microscopy at 37°C, the restrictive temperature (Fig. 1A). The temperature-sensitive *CDC28* allele was used to observe cells in G1 as opposed to using the mating pheromone alpha-factor, as the latter treatment causes Kar3Cik1 to lose its nuclear localization sequence in order to function in the cytoplasm and to mediate karyogamy (Allingham et al., 2007; Gibeaux et al., 2006; Meluh and Rose, 1990). Cells arrested in G1 had the stereotypical bright nuclear microtubule bundle and long cytoplasmic MTs (cMTs). Of all cells, 77.3% contained a single GFP-Kar3 spot near the SPB (Fig. 1B). When cells were arrested in S phase, the majority had short spindles and, of these, 96.4 % had a bright GFP-Kar3 spot near each SPB. GFP-Kar3 also appeared along the spindle and as a lobed haze typical of kinetochore localization (Xiangwei et al., 2000). For 97.3% of cells arrested in metaphase, short spindles showed GFP-Kar3 localization along their length and SPBs demonstrating greater GFP-Kar3 fluorescence intensity than the spindle. The remaining 2.7% of cells had GFP puncta only near the SPBs. During anaphase, GFP-Kar3 was observed near the SPBs along the length of the spindle and as a lobed haze close to the SPB, again likely to be reflecting kinetochore localization in 97.1% of cells.

We quantified the GFP-Kar3 signal along spindles in S phase-, metaphase- and anaphase-arrested cells (Fig. 1C). Fluorescence was brightest along the spindle during metaphase; less intensity was observed during S phase, despite spindles being of similar length. In anaphase, fluorescence intensity was greatest at or near the poles. Although bright puncta were observed along the length of individual extended spindles, their positions were irregular and did not favor any particular regions.

We then determined the localization of both distinct Kar3 heterodimers by tagging either Cik1 or Vik1 with mScarlet-I (hereafter referred to as mScarlet) (Bindels et al., 2017) paired with *GFP-TUB1* and arrested the cells by using conditional *cdc* alleles, as above. Interestingly, we found that Cik1-mScarlet was not detectable in G1-arrested cells (Fig. 1D). By contrast, 89.3% of Vik1-mScarlet-expressing cells had a single punctum near the cMT origin (Fig. 1E), indicating that the single spot seen at the SPB in G1-arrested GFP-Kar3 cells only comprised Kar3Vik1 heterodimers. At S phase, Cik1 was only present on short spindles (34.4% of cells), near SPBs (25.0% of cells) or on both structures (15.6% of cells). In contrast, Vik1 was always observed near the spindle poles, presumably in the cytoplasm. Upon arrest in metaphase, Cik1 localization slightly shifted to a bi-lobed configuration along short spindles with bright localization at the spindle ends in 66.1% of cells – a pattern typical for proteins associated with kinetochores (Gillett et al., 2004). Cik1-mScarlet on spindles but not SPBs was observed in 29.7% of cells, and 4.2% of cells only showed SPB localization. As in S phase-arrested cells, Vik1 was always found on SPBs. Typical for kinetochores, Cik1 localization was observed along the extended spindles and as a haze near SPBs within the nucleus in 89.4% of the anaphase-arrested cells. Only 10.6% of these cells showed bright puncta adjacent to SPBs together with spindle localization. Vik1 localization during anaphase was very similar to that observed during S phase and metaphase, and a bright spot in the cytoplasm was always observed near the SPB.

To fully quantify these localization patterns, we also measured the relative fluorescence intensities of either Cik1-mScarlet or Vik1-mScarlet and compared them to the relative fluorescence intensity of GFP-Tub1 along the spindle in cells arrested in metaphase (Fig. S1). The peak fluorescence of Cik1-mScarlet coincided with, or was within, the peak of GFP-Tub1 fluorescence, which is presumably the SPB. In contrast, fluorescence levels for Vik1-mScarlet peaked outside those for tubulin, dropping off completely thereafter. This quantification indicates that Cik1, with its nuclear localization sequence, is found inside the nucleus, whereas Vik1 is excluded and stays solely on the cytoplasmic face of the SPB. These observations confirm that the location of Kar3Cik1 changes in a programmed manner throughout the vegetative cell cycle. By contrast, Kar3Vik1 is localized at the SPB during all stages of the cell cycle.

**Fig. 2.**
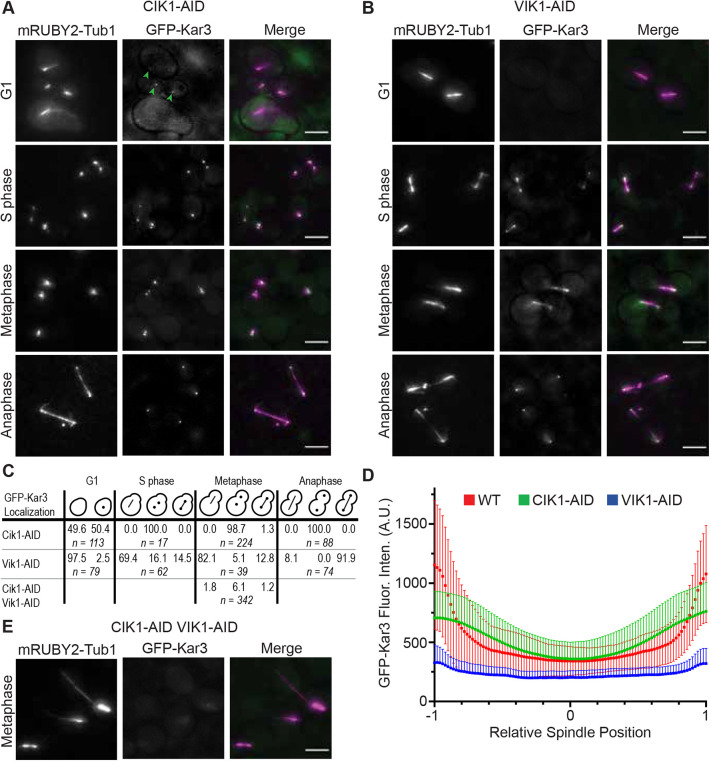
**Kar3 accessory proteins direct Kar3 localization and MT morphology.** (A,B) GFP-Kar3 (green) localization and mRUBY2-Tub1 (magenta) morphology in cell cycle-arrested cells depleted of Cik1-AID (A) or Vik1-AID (B). Arrowheads indicate GFP-Kar3 near SPBs. (C) Percentage of cells with GFP-Kar3 localization at SPB, spindle or both structures at each point of the cell cycle. *n*=number of cells counted. (D) Line scans of GFP-Kar3 fluorescence intensity along spindles in WT, Cik1-depleted or Vik1-depleted cells that had been arrested in metaphase. *n*=50. Error bars indicate the mean±s.d. A.U., arbitrary units. (E) Localization of GFP-Kar3 and mRUBY2-Tub1 in metaphase-arrested cells depleted of both Cik1 and Vik1. Scale bars: 5 µm.

Next, we wanted to investigate the roles of Cik1 or Vik1 in MT organization and assembly in cells and for Kar3 localization throughout the cell cycle. To achieve this, we either introduced a *CIK1-AID* or *VIK1-AID* allele into the *GFP-KAR3 mRUBY2-TUB1 cdc^ts^* strains used above. The *AID* motif is an auxin-inducible degron that triggers proteolysis of the tagged protein in the presence of 3-indole acetic acid (3-IAA) (Nishimura et al., 2009; Morawska and Ulrich, 2013). The AID-tagged proteins were depleted for 30 min from cells kept at the restrictive temperature (37°C) for 3 h. Western blot analysis showed that this was sufficient time for Kar3 and Cik1 proteins to be depleted by 90% and for Vik1 to be depleted by 75% (Fig. S2A). The amount of GFP-Kar3 was unchanged when these accessory proteins were depleted, indicating that it is the individual tagged proteins being degraded before interacting as a heterodimer (Fig. S2B). These strains gave us tight control when proteins levels were lowered and, therefore, helped to prevent unwanted secondary effects due to complete absence of these proteins throughout the lifetime of a cell. When cells were mounted on coverslips, the pre-warmed medium also contained 3-IAA to continue protein depletion during the observation time. Cells arrested in G1 and depleted of Cik1 retained a single spot of GFP-Kar3 at SPBs (50.4% of cells) as was seen in non-depleted cells (Fig. 2A, arrowheads). When S-phase-arrested cells were depleted of Cik1, GFP-Kar3 was never observed on nuclear MT structures. In metaphase-arrested cells depleted of Cik1, GFP-Kar3 was absent from the spindle but strong fluorescence of GFP-Kar3 puncta was observed in the cytoplasm, presumably near the SPB, in 98.7% of cells. Similarly, when Cik1 was depleted during late anaphase, GFP-Kar3 was found only at the SPBs and not along the extended spindle.

Effects of Vik1 depletion were consistent with the difference in heterodimer localization (Fig. 2B). During G1 arrest, all but 2.5% of Vik1-depleted cells lacked the GFP-Kar3 spot seen at the SPB in WT and Cik1-AID cells (Figs 1A and 2A-C). GFP-Kar3 was only observed near SPBs in 30.6% of S phase-arrested cells depleted of Vik1, instead of 98.2% observed in WT cells. During metaphase and upon depletion of Vik1, detectable levels of GFP-Kar3 at SPBs were only found in 17.9% of cells, whereas 100% of WT cells showed bright GFP-Kar3 fluorescence near SPBs. In anaphase-arrested cells depleted of Vik1, GFP-Kar3 was absent from the cytoplasm but a cloud of fluorescence was present in the nucleus near the SPBs, consistent with kinetochore localization.

To confirm the change in Kar3 localization during metaphase upon depletion of each of the accessory proteins, we quantitatively analyzed the fluorescence intensity of the GFP-Kar3 signal along the spindle (Fig. 2D). The values for WT cells in this figure are the same as those presented in Fig. 1C as reference. Noticeable are the strong peaks at the spindle ends, with uniform intensity along its length. When Cik1 was depleted, the intensity peaks at the poles decreased. When Kar3Cik1 was absent, the overall length of spindles decreased and, in some cases, the spindles were broken. These effects made it difficult to determine where fluorescence from the SPB ended and that from the spindle began. Upon Vik1 depletion, peaks at the spindle ends nearly disappeared, suggesting that most of Kar3 localization to SPBs during metaphase is due to Vik1.

We next determined whether Kar3 is able to interact with MTs in cells when both binding partners, Cik1 and Vik1, are absent. For this, we created a strain that expresses both *CIK1-AID* and *VIK1-AID* alleles together with *GFP-KAR3* and *mRUBY2-TUB1*. This strain also contains the *cdc23-1* allele, which enabled us to arrest cells in metaphase. When these cells were arrested and the Kar3-binding partners depleted as above, GFP-Kar3 was detected near the SPBs in 3.0% of cells or, when in association with the nuclear MTs, in only 7.3% of cells (Fig. 2E) – a drastic reduction compared to levels in WT cells. To evaluate the opposite possibility, i.e. that Cik1 or Vik1 relied on Kar3 for their interaction with MTs, we introduced the *KAR3-AID* allele into our *GFP-TUB1*-expressing strains co-expressing either *CIK1* or *VIK1* tagged with mScarlet. Upon depletion of Kar3 from these strains, Cik1- or Vik1-mScarlet signals became diffuse throughout the cell, without any puncta even at SPBs (Fig. S3). These data demonstrate that only heterodimers and not individual Kar3, Cik1 or Vik1 proteins, can associate with MTs *in vivo*.

### Kar3Cik1 controls spindle length whereas Kar3Vik1 affects cytoplasmic MTs

**Fig. 3.**
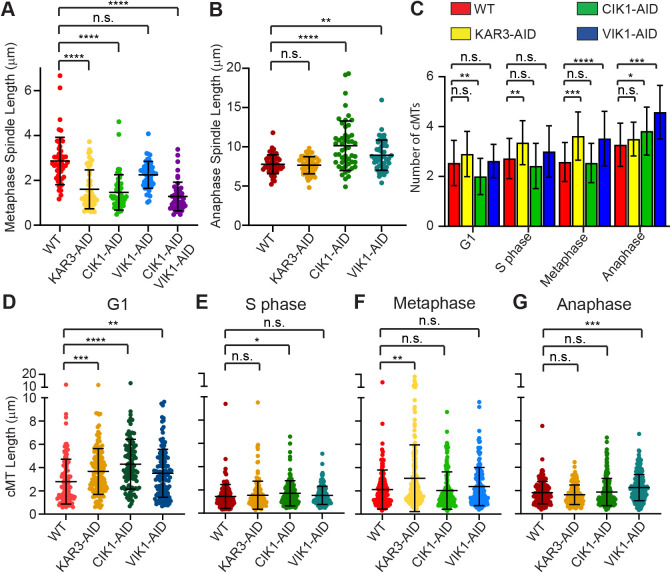
**Spindle lengths, cMT number and cMT lengths in yeast cells depleted for Kar3/Cik1/Vik1.** (A,B) Spindle lengths of yeast WT cells, cells depleted of Kar3, Cik1 or Vik1, or depleted for both Cik1 and Vik1 – all arrested in metaphase (A) or late anaphase (B). *n*=50. Error bars indicate the mean±s.d. Statistical significance was determined by one-way ANOVA using the Kruskal–Wallis test. ns, not significant; *P<0.05, **P<0.01; ***P<0.0005; ****P<0.0001. (C) Average number of cMTs per cell during the cell cycle. Analyzed were WT cells and cells depleted of Kar3, Cik1 or Vik1. *n*=50. (D–G) Quantification of cMT lengths obtained from WT cells, and from cells depleted of Kar3, Cik1 or Vik1, arrested at G1 (D), S phase (E), metaphase (F) or anaphase (G). *n*=50. Error bars indicate the mean±s.d. Statistical significance for data shown in panels C–G was determined by one-way ANOVA using the Kruskal–Wallis test and by comparing depleted cells to those of WT. ns, not significant; *P<0.05; **P<0.01; ***P<0.0005; ****P<0.0001. Red, WT; yellow, Kar3 depleted; green, Cik1 depleted; blue, Vik1 depleted; purple, Cik1 and Vik1 depleted.

Previous studies have described MT morphology phenotypes for *kar3Δ* cells that typically included longer, more numerous cMTs (Huyett et al., 1998; Saunders et al., 1997b) and short or broken spindles during metaphase (Hoyt et al., 1993; Saunders et al., 1997a). Similar phenotypes have been observed in single-depletion cells (Kornakov et al., 2020), which we here set out to define quantitatively according to cell cycle stage. Most notable were differences in the spindle lengths between cells. To measure spindle lengths, *GFP-TUB1*-expressing cells were arrested in metaphase or anaphase, followed by depletion of Kar3, Cik1 or Vik1 by using the AID system. Cells were then observed during cell cycle arrest and depletion conditions. Measurement of spindles was done at the first time point. In *KAR3-AID* and *CIK1-AID* cells arrested in metaphase spindles formed; however, during time-lapse observation, intact spindles broke in 29.8% of Kar3-depleted and 36.5% in Cik1-depleted cells. In Kar3- and Cik1-depleted cells the mean distance between SPBs of intact spindles was much shorter compared to that in non-depleted cells (1.6±0.9, 1.4±0.8 vs 2.9±1.1 μm, respectively) (Fig. 3A). Spindles in Vik1-depleted metaphase-arrested cells were similar in length to spindles in WT cells (2.3±0.6 μm) and they did not break. To further characterize this phenotype, we utilized a strain in which Cik1 and Vik1 could simultaneously be depleted. The spindles in these metaphase cells were on average 1.3±0.6 μm long and resembled those of Kar3-AID or Cik1-AID cells in the presence of 3-IAA, i.e. when Kar3 or Cik1 were depleted. The above observations are consistent with those made in previous studies of spindles in *kar3*Δ*, cik1*Δ or *vik1*Δ cells (Hepperla et al., 2014), which indicates that Kar3Cik1 is needed to balance the forces along interpolar MTs. As for cells arrested in late anaphase and depleted of Kar3, their spindle length was similar to that in WT cells (7.6±1.1 vs 7.8±1.2 μm, respectively) (Fig. 3B), suggesting that Kar3 heterodimers are not necessary for maintaining elongated spindles. However, the picture becomes more complex when either Cik1 or Vik1 is depleted. Upon depletion of Cik1, spindles were longer than those in WT (10.1±3.2 μm) and, occasionally, exceeded the length of the cell, bending around the cell interior (Fig. 3B). Cells arrested in late anaphase and depleted of Vik1 also had longer spindles (8.9±1.9 μm on average). Since these two accessory proteins are in different cellular compartments and since the magnitude of this increase is different, the mechanism underlying an increase in spindle length is likely to be different.

Previous research has found that cells lacking functional Kar3 have more cMTs and that these cMTs are longer on average than those observed in WT cells (Huyett et al., 1998; Saunders et al., 1997b). We quantified the number and length of cMTs in strains that express *GFP-TUB1*, an AID-tagged protein as well as temperature-sensitive *CDC*-mutant alleles that allows arrest at different cell cycle stages. We found that cells depleted of Kar3 had an increased number of cMTs in S phase and metaphase (Fig. 3C). Upon depletion of Cik1 in G1-arrested cells, the number of cMTs decreased (Fig. 3C). However, Cik1 depletion only significantly increased cMT numbers in anaphase but not in in S phase or metaphase. In cells depleted of Vik1, the number of cMTs increased in metaphase and anaphase, suggesting a greater role for Kar3Vik1 in the regulation of cMT numbers during mitosis (Fig. 3C).

Surprisingly, the length of cMTs in Kar3-, Cik1- or Vik1-depleted cells was almost indistinguishable from that of cMTs in WT cells during all cell cycle stages – except during G1, when depletion of either protein complex increased the length of cMTs (Fig. 3D–F). Interestingly, only Vik1 depletion resulted in increased cMT length in late anaphase (Fig. 3G), which contradicts previous reports of elongated cMTs in *kar3Δ* cells. Therefore, perhaps the increased length of cMTs observed by us during G1 or late anaphase in cells with compromised Kar3Vik1 persists throughout the cell cycle if Kar3Vik1 function is not restored.

### Distinct contribution of Kar3 heterodimers to MT dynamics varies with the cell cycle stage

Knowing that Kar3Cik1 and Kar3Vik1 have distinct subcellular localizations, we set out to compare their biochemical activities. Previous studies of Kar3 revealed its potential MT depolymerase activity and possible interactions with MAPs that are known to regulate MT dynamics (Endow et al., 1994; Sproul et al., 2005; Tirnauer et al., 1999). We, therefore, set out to determine the effects of the two different Kar3 heterodimers on MT dynamics by using our previously developed reconstitution assay (Bergman et al., 2019). This assay enables us to observe the dynamics of single MTs in the presence of total soluble protein lysate.

By using this reconstitution assay, we have previously shown that the effects Kar3Cik1 and Kar3Vik1 heterodimers have on MT dynamics are substantially different (Bergman et al., 2019). Lysates created from asynchronous *S. cerevisiae* WT cultures do not assemble MTs, and the lack of Cik1 in lysate from asynchronous cells did not change this result. In contrast, cell lysates lacking Vik1 readily polymerize MTs, similar to asynchronous lysates lacking either Kip3 or Kar3 (Bergman et al., 2019). Therefore, we further investigated the contribution of each heterodimer to the regulation of MT dynamics and with greater precision, by utilizing our *GFP-TUB1*-expressing strains that, within their C-terminus, also express *KAR3*, *CIK1* or *VIK1* tagged to the AID motif. Each of these strains also contain a temperature-sensitive allele of the *CDC* gene enabling us to arrest cells at different cell cycle stages. We then prepared whole-cell lysates from arrested and depleted yeast cell cultures, and assayed their MT dynamics.

**Fig. 4.**
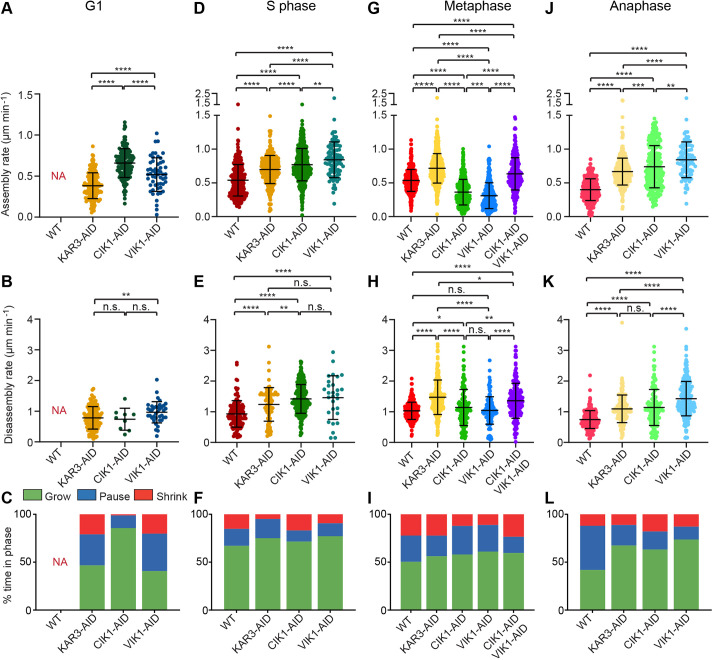
**Kar3, Cik1 and Vik1 have different effects on reconstituted MT dynamics.** (A–L) Quantification of reconstituted MT dynamics in lysates from cells arrested in G1 phase, S phase, metaphase or anaphase. Differences in MT assembly rates (A,D,G,J), disassembly rates (B,E,H,K) and their growth parameters – i.e. MT growth, growth stagnation (pause) and shrinkage (C,F,I,L) – were seen between lysates of WT cells, and lysates of cells depleted for Kar3, Cik1, Vik1, or Cik1 and Vik1. Error bars indicate the mean±s.d. Statistical significance was determined by pairwise Student's *t*-test, with ns, not significant; *P<0.05; **P<0.01; ***P<0.0005; ****P<0.0001. NA, no assembly.

Unlike lysates from WT cells arrested in G1, in which MTs do not assemble, lysates depleted of Kar3, Cik1 or Vik1 can each assemble dynamic MTs. MTs in lysates made from G1-arrested Kar3-depleted cells assembled MTs at a rate of 0.38±0.16 µm min^−1^ (Fig. 4A), with a disassembly rate of 0.78±0.37 µm min^−1^ (Fig. 4B). MTs assembled faster (0.66±0.17 and 0.52±0.20 µm min^−1^, respectively) in Cik1-depleted and Vik1-depleted lysates. MTs disassembled at a similar speed in Cik1-depleted lysates or faster when Vik1 was depleted (0.74±0.36 and 0.97±0.35 µm min^−1^, respectively) (Fig. 4B). Globally, MTs in Kar3-depleted lysates grew 46.6% of the time, shrank 21% of the time and paused for the remaining 32.4% of the time (Fig. 4C). MTs in Cik1-depleted lysates grew 85.6% of the time and only paused 13.2% of the time. Surprisingly, MTs in Vik1-depleted lysates only grew 40.7% of the time and shrank 20.2% of the time, resembling the distribution seen in Kar3-depleted lysates. The frequency of catastrophe in Kar3- and Vik1-depleted lysates was about 10-fold higher than that of MTs in the Cik1-depleted lysate (2.7 or 3.1 vs 0.3×10^−3^ events s^−1^) (Table 1). These data suggest that, in G1, Kar3 heterodimers either behave as MT depolymerases or they prevent MT polymerization via an unknown mechanism. This conclusion is in agreement with previous data (Huyett et al., 1998; Saunders et al., 1997b; Tirnauer et al., 1999) and our own cellular imaging (Fig. 3D), which show an increase in the number and length of cMTs in unbudded cells lacking Kar3 heterodimers. These data further suggest that Kar3 heterodimers are the main depolymerase or catastrophe factor that prevent MTs from growing in G1-arrested lysates, as we observed during this study and in our previous work (Bergman et al., 2019).

**Table 1.**
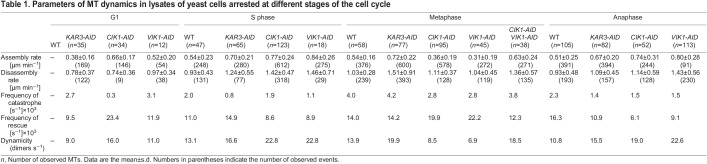
Parameters of MT dynamics in lysates of yeast cells arrested at different stages of the cell cycle

In S phase-arrested lysates depleted of Kar3, Cik1 or Vik1, MT assembly and disassembly rates both increased relative to rates in lysates from S phase-arrested WT cells (Fig. 4D,E). The overall proportion of time when MTs were growing increased for all three lysates relative to the proportion in lysates from WT cells (Fig. 4F). Thus, in S phase-arrested lysates, Kar3 heterodimers appear to be responsible for muted assembly and disassembly rates. Interestingly, only in Kar3- and Vik1-depleted lysates the frequency of catastrophe was significantly lower than in WT lysates (Table 1). This result was reflected in the disassembly time of MTs in these lysates (Fig. 4F). Thus, Kar3Vik1 acts to promote catastrophes during S phase.

Upon acute Kar3, Cik1 or Vik1 depletion via the AID degron system in lysates of metaphase-arrested cells, the role of Kar3 heterodimers in the regulation of MT dynamics appears similar in our assay (Fig. 4G,H). When Kar3 was depleted from metaphase lysates, MT assembly (0.54±016 vs 0.72±0.22 µm min^−1^) and disassembly (1.03±0.28 vs 1.51±0.91 µm min^−1^) rates both increased relative to rates in WT lysates (Fig. 4G,H). Interestingly, MT assembly rates in Cik1- and Vik1-depleted metaphase lysates were lower than those in WT lysates (Fig. 4G), whereas MT disassembly rates in metaphase lysates depleted for Cik1 and Vik1 remained similar to those in WT lysates (Fig. 4H). MTs in Kar3-depleted cell lysates spend less time paused (i.e. not changing in length) than those in WT lysates (21% vs 27%, respectively; Fig. 4I, compare blue ‘pause’ sections). MTs in Cik1- or Vik1-depleted lysates, however, exhibit a frequency of catastrophe that is 30% less compared to that of MTs in WT lysates (Table 1). This reduced catastrophe rate leads to a decrease in the time MTs spend disassembling compared to that of MTs in WT lysates (Fig. 4I). We further investigated this phenomenon by using the strain expressing both AID-tagged *CIK1* and AID-tagged *VIK1*. In lysates created from cells of this strain arrested in metaphase, MT dynamics were similar to those in Kar3-depleted lysates in that both assembly and disassembly rates were faster than in WT lysates, and in that the dynamicity – defined as the number of tubulin dimers exchanging at an MT end per second – and the frequency of catastrophe were increased (Fig. 4G,H, Table 1). These data suggest that, during metaphase, Kar3Cik1 and Kar3Vik1 regulate MT dynamics by similar mechanisms *in vivo* but that these protein complexes are normally separated into cytoplasmic and nuclear compartments. In our assay, these effects are likely to become synergistic as a result of removing the nuclear envelope barrier that separates Kar3Cik1 and Kar3Vik1 in cells.

The apparent functions of Kar3Cik1 and Kar3Vik1 heterodimers diverged most sharply in anaphase. When MT dynamics parameters were measured in lysates of WT, *KAR3-AID*, *CIK1-AID* or *VIK1-AID* cells that had been arrested in anaphase, a complex picture emerges. All depletions increased MT assembly as well as disassembly rates (Fig. 4J,K). And, compared to MTs in WT lysates, all depletions decreased the time MTs spend paused (Fig. 4L), making for highly dynamic MTs. However, in lysates depleted for Kar3 and Cik1, MTs had similar disassembly rates and catastrophe frequency to each other. MTs in Vik1-depleted lysate were even faster and more dynamic (Table 1). These data suggest that Kar3 heterodimers normally act to increase MT pausing in anaphase, and that Kar3Vik1 has a stronger effect on MT dynamics than Kar3Cik1. In total, these results mirrored the MT behavior observed in *S. cerevisiae* of late anaphase cells, such that cMTs (Zucca et al., 2022) and kinetochore MTs (kMTs) (Higuchi and Uhlmann, 2005) were kept short and stable in their respective cellular compartments.

### Kar3 heterodimers have disparate associations with MTs

**Fig. 5.**
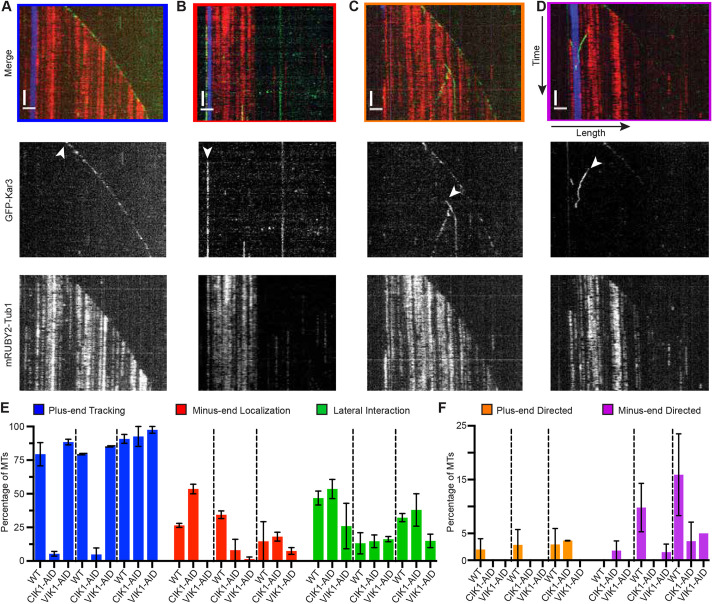
**GFP-Kar3 heterodimers associate differently with reconstituted MTs.** Dynamic MTs were reconstituted from lysate of cells expressing *GFP-KAR3* (green) and *mRUBY2-TUB1* (red). These MTs were adhered to coverslips by using HiLyte647-labeled seeds (blue). (A–D) Kymographs representing spatial position of GFP-Kar3 (x-axes) over time (y-axes) to observe motor association with and translocation along the MT. Horizontal bars: 2 µm; vertical bars 3 min. White arrowheads indicate GFP-Kar3 molecules that track the growing plus-end of an MT (A), localize to the minus-end of an MT seed (B), move towards the plus-end of a growing MT (C), or move towards the minus-end of an MT (D). (E,F) Percentage of observed MTs that display non-motile (E) and motile (F) interactions with GFP-Kar3. Dashed lines within each set of colored bars separate the cell cycle stages S phase, metaphase and anaphase. Color outlines of merged images in A–D correspond to sets of colored bars in E and F. Error bars indicate the mean±s.e.m.

To determine if the two Kar3 heterodimers have dynamic profiles on MTs independent of intracellular localization, we created soluble, whole-cell lysates from strains expressing *GFP-KAR3* and *mRUBY2-TUB1* that could be arrested at different cell cycle stages using a temperature-sensitive *CDC23* allele. From lysates created from metaphase-arrested cells, we observed four different classes of GFP-Kar3 interactions with MTs polymerized from HiLyte647-labeled tubulin seeds: (1) tracking the plus-end of a growing MT (Fig. 5A), (2) interaction with the stable minus-end of a seed (Fig. 5B), (3) movement along the MT lattice towards the plus-end (Fig. 5C), and (4) movement along the lattice towards the MT minus-end (Fig. 5D).

We next set out to determine which behavior each heterodimer exhibited during different cell cycle phases. We analyzed soluble lysates lacking either Cik1 or Vik1 by using the AID system and counted how many times these different interactions were observed (Fig. 5E,F, Table 2). Only MTs comprising detectable GFP-Kar3 were quantified, i.e. ∼50% of all observable MTs, even after taking steps to encourage these interactions (see Materials and Methods). In WT lysates made from cells arrested in S phase, 79.4±8.6% of MTs had GFP-Kar3 on their growing plus-end. This dropped to only 5.4±1.8% in Cik1-depleted lysates and remained high (88.5±2.1% of MTs) in Vik1-depleted lysates, implicating Cik1 in plus-end tracking during S phase. Interestingly, the amount of GFP-Kar3 found on the minus-ends of seeds increased in Cik1-depleted lysates (53.6±3.6%) compared to WT (26.5±1.5%) and this localization was absent in Vik1-depleted lysates. This observation matched our *in vivo* data (Fig. 2B,C) in that GFP-Kar3 did not have strong SPB-associated signals in 69.4% of S phase-arrested Vik1-depleted cells, whereas 98.2% of WT and 100% of *CIK1-AID* cells had GFP-Kar3 at SPBs. Moreover, static interaction of GFP-Kar3 along the MT lattice dropped from around 50% of observed MTs in WT or Cik1-depleted lysates to only 26.0±16.9% of MTs in Vik1-depleted lysates.

**Table 2.**
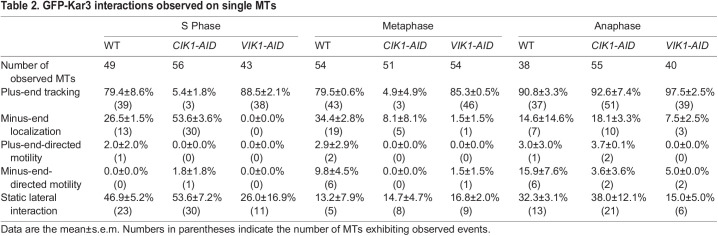
GFP-Kar3 interactions observed on single MTs

In metaphase-arrested lysates, GFP-Kar3 was observed tracking the plus-end in 79.5±0.6% of observed MTs. This number dropped to only 4.9±4.9% of MTs when Cik1 was depleted. However, GFP-Kar3 was observed tracking plus-ends in 85.3±0.5% of MTs in Vik1-depleted lysates. These observations demonstrate that Cik1 is also necessary for Kar3 to track plus-ends during metaphase. In WT extracts, GFP-Kar3 interacted with stabilized MT minus-ends in 34.4±2.8% of MTs but fewer (8.1±8.1% of MTs) instances were observed in Cik1-depleted lysates and even fewer (1.5±1.5%) in Vik1-depleted lysates. This observation matches our *in vivo* data showing that GFP-Kar3 localized to both sides of the SPB, from which yeast MTs emanate. GFP-Kar3 no longer localized to the SPB on the nuclear or cytoplasmic side of the nuclear envelope when the corresponding Kar3 accessory protein was depleted. We observed minus-end-directed motility of Kar3 on 9.8±4.5% of MTs in lysates from WT cells. Motility was not observed in Cik1-depleted lysates. Only one out of 54 MTs (1.5±1.5%) in Vik1-depleted lysates had motors moving in minus-end direction. These data suggest that the minus-end-directed motility observed in our assay depends on both Kar3 heterodimers being present in metaphase lysates.

During anaphase, activities of the two heterodimers appear to diverge. Surprisingly, Cik1 depletion did not prevent GFP-Kar3 from tracking the plus-end (90.8±3.3% WT and 92.6±7.4% *CIK1-AID*) or from translocating towards the plus-end along the lattice. In contrast, Vik1 depletion decreased the number of MTs with GFP-Kar3 on the minus-end, GFP-Kar3 molecules with plus-end-directed motility, and static interactions of GFP-Kar3 along MT lattices. Additionally, diffuse GFP-Kar3 signals were now observed along the lengths of MTs in Cik1-depleted lysates during anaphase (Fig. S4). Again, minus-end-directed motility was compromised when either Cik1 or Vik1 was depleted. These and our other results point to an important role for Vik1 in controlling the number and length of cMTs during anaphase, by directing localization and translocation of Kar3.

Finally, we took this opportunity to measure the rate of Kar3 minus-end-directed motility. We determined the rate of movement in a manner similar to that used to calculate the rate of MT assembly or disassembly. In our assay, GFP-Kar3 moved towards the minus-end at a rate of 0.64±0.27 μm min^−1^ (10.6 nm s^−1^) in metaphase-arrested cell lysates, which is much slower than previously reported *in vitro* rates (Allingham et al., 2007; Chu et al., 2005; Endow et al., 1994; Mieck et al., 2015). However, this rate is similar to the *in vivo* rate reported for captured centromeres that move along the lattice of kMTs towards the minus-end in a Kar3-dependent manner (Tanaka et al., 2007). Kinesin-14 motors are typically non-processive (Case et al., 1997; Foster and Gilbert, 2000). The processivity of GFP-Kar3 in our assay was 0.88±0.57 μm, which is ∼6× lower than that reported for Kar3Cik1 (5.2 μm) (Mieck et al., 2015). The latter study also found that Kar3Cik1 processivity is highly dependent on ionic strength (Mieck et al., 2015). Our reconstitution assay might more closely approximate cellular conditions, thereby accounting for this difference.

## DISCUSSION

In this work, we used *in vivo* studies and our previously developed reconstitution system (Bergman et al., 2019) to examine in detail localization and function of the budding yeast kinesin-14 Kar3 as well as of its two binding partners Cik1 and Vik1 through the cell cycle. During mitotic growth these three proteins form the two distinct heterodimers Kar3Cik1 and Kar3Vik1. During vegetative growth, the former is found exclusively in the nucleus, whereas the latter is found exclusively in the cytoplasm (Fig. 1; Fig. S1) (Manning et al., 1999). By individually expressing these three proteins along with tubulin as fluorescent fusion proteins in cells carrying temperature-sensitive alleles of cell-cycle-control genes, we were able to describe the localization of each heterodimer during four distinct cell cycle stages. Then, by selectively disrupting one heterodimer at a time by using an inducible degradation system for either Cik1 or Vik1, we were able to relate cell cycle-regulated localization of each heterodimer *in vivo* to biochemical activities *in vitro*.

### Cik1 and Vik1 impart distinct functionality to Kar3

Using our reconstitution system, we found that – although Kar3Cik1 and Kar3Vik1 are similar in their predicted structure (Chu et al., 2005; Manning et al., 1999) – they distinctly contribute to regulate MT dynamics at certain stages of the cell cycle. Furthermore, using three-color TIRF microscopy, we observed complex Kar3 behaviors, i.e. motility along MTs, static interactions with either the minus-end or the lattice, and tracking of dynamic plus-ends. Interestingly, these activities were depending on which binding partner was associated with Kar3 and on the stage of the cell cycle.

Our previous reconstitution studies provide evidence for differences in the functions of the two Kar3 heterodimers (Bergman et al., 2019). Some caveats exist within our reconstitution system, such as the mixing of nucleoplasm and cytoplasm, the lack of spatial cues, and that we observed only single MTs (Bergman et al., 2019) but, despite these caveats, we believe outstanding questions could be addressed in new ways. Although WT lysates from asynchronous cells do not assemble MTs, similar lysates from *vik1Δ* cells – but not *cik1Δ* cells – do assemble MTs (Bergman et al., 2019), an observation that motivated us to address unsettled questions regarding Kar3 function and regulation in this current study.

Our *in vitro* studies found that, when Cik1 or Vik1 were depleted, frequency of catastrophe decreases and the overall dynamicity of MTs increases because assembly and disassembly rates increase (Fig. 4, Table 1). Interestingly, the Kar3Cik1 heterodimer appears to control the number of catastrophes during G1, whereas Kar3Vik1 has a more-prominent role during S phase. Both heterodimers contribute to dynamicity and frequency of catastrophe during mitotic phases.

Previous studies have indicated that Kar3Cik1 catalyzes MT depolymerization (Endow et al., 1994; Sproul et al., 2005), whereas Kar3Vik1 does not (Allingham et al., 2007). A number of not-yet-tested mechanisms have been proposed to explain how Kar3 destabilizes the MT plus-end as it translocates towards the minus-end (Hou and Wang, 2010; Ogren et al., 2022). Our motor-tracking dynamics data suggest a higher level of functional and mechanistic complexity. Although we observed GFP-Kar3 molecules tracking disassembling plus-ends, GFP-Kar3 was also found on growing plus-ends and moving along the lattice towards the minus-end without causing catastrophes (Fig. 5, Table 2). Our observation that Kar3 differs in depolymerase activity during different cell cycle stages (Fig. 4F,I,L, Fig. 5E) indicates that more work is needed to fully elucidate its mechanism of controlling MT dynamics and its regulation. The finding that Kar3 depletion increases both assembly and disassembly rates raises more mechanistic questions for future studies. Budding yeast has an additional known MT depolymerase, the kinesin-8 Kip3 (Gupta et al., 2006). Based on the data presented here and in our previous work, in which we quantitatively analyzed MT dynamics in the absence of one or both of these depolymerases, it seems possible that removal of these proteins from MTs creates new opportunities for other MAPs to affect assembly and disassembly rates (Bergman et al., 2019). The budding yeast EB1 homolog Bim1 is responsible for promoting MT growth (Blake-Hodek et al., 2010; Wolyniak et al., 2006). One possibility is that, when one of its binding partners – e.g. Kar3Cik1 – is absent, more Bim1 is available to increase growth rates (Kornakov et al., 2020). It might also be that Kar3 heterodimers induce catastrophes but modulate MT disassembly rate once a catastrophe has occurred.

Locomotion of Kar3 heterodimers in either direction on MTs in our *in vitro* motor-tracking assay was rare and never observed on cMTs in our time-lapse imaging of live cells. The plus-end-directed motility we observed at all cell cycle stages *in vitro* varied in velocity and duration. Also, its mechanism is unclear. In contrast, the minus-end-directed motility we observed in metaphase lysates was more uniform in velocity, matching the speed of captured kinetochores being transported along single kMTs in cells in an inducible chromosome capture system (Tanaka et al., 2005). Our calculated minus-end-directed velocity of 0.64±0.27 μm min^−1^ was ∼7-fold slower than velocities (4.6 μm min^−1^) reported by others for Kar3Cik1 (Mieck et al., 2015). Mieck and colleagues reported that Kar3Vik1 moves at speeds approaching 14 μm min^−1^, a speed that we would not observe with our sampling rate. Such a high motility rate might explain our failure to observe Kar3Vik1 movement along cMTs in live cells.

### Differential cell cycle regulation of Kar3 heterodimers

By combining cell cycle arrests and acute, targeted depletion of accessory proteins, we showed at a subcellular level where Kar3Cik1 and Kar3Vik1 function on MTs throughout the cell cycle. Fluorescently tagged Kar3 has been used previously to ascertain where Kar3 heterodimers are located in cells (Gardner et al., 2008; Gibeaux et al., 2006; Maddox et al., 2003; Mieck et al., 2015), but studies of this fusion have not focused on how localization transitions from one cell cycle stage to the next. Recent work has shown that absence of Cik1 or Vik1 changes Kar3-GFP localization on anaphase spindles (Mieck et al., 2015). We confirmed these previous observations and extended this analysis to different cell cycle stages (Fig. 1A). Moreover, we determined which Kar3 heterodimer populations contain Cik1 or Vik1 by tagging one at a time to mScarlet (Fig. 1D,E). These data, combined with analysis of strains expressing the degron-tagged version of an accessory protein together with GFP-Kar3, independently confirmed that Kar3Cik1 resides on the nuclear face of SPBs, along the spindle and on kMTs (Fig. 2A; Fig. S1B), and that Kar3Vik1 is on the cytoplasmic face of SPBs (Fig. 2B; Fig. S1C). We also confirmed that interaction of Kar3 with MTs is dependent upon heterodimer formation (Fig. 2E) and that the accessory proteins cannot bind MTs in the absence of Kar3 (Fig. S3) (Manning et al., 1999).

Our *in vivo* localization data are consistent with our *in vitro* observations. The most common Kar3-MT interaction we observed on single MTs was plus-end tracking, which was highly, but not completely, dependent on Cik1 (Table 2). This observation aligns well with Kar3Cik1 localization on kMTs in cells (Fig. 1). Association of Kar3 with MT minus-ends in our assay was the second most common observed localization. This minus-end association was more dependent on Vik1 than Cik1 (Fig. 5). Consistently, SPB staining in cells was only slightly diminished in Cik1-depleted cells but was almost absent in the Vik1-depleted cells.

Previous analysis of *kar3Δ* phenotypes revealed an increase in cMT number and length (Huyett et al., 1998; Saunders et al., 1997b). We also observed an increase in cMT numbers for Kar3- and Vik1-depleted cells at various cell cycle stages (Fig. 3C). However, average cMT length only increased in G1 in cells depleted of Kar3, Cik1 and Vik1, in metaphase for cells depleted of Kar3, and in anaphase for cells depleted of Vik1 (Fig. 3D-F). Our results may differ from those reported previously because of one or more of several differences in experimental design. Instead of observing asynchronous cells carrying gene disruptions, we observed arrested cells depleted of a protein of interest after using a degron tag. Perhaps the increase in cMT length reported in earlier studies was the result of the absence of a protein over multiple cell cycles because a null-mutant was used instead of acute, degron-driven depletion. We observed that depletion of Kar3 and Cik1 both lead to shorter metaphase spindles that collapse or break as mitosis progresses. This suggests that Kar3Cik1 is necessary for building a spindle of appropriate length and for maintaining the necessary opposing force across the spindle (Figs 2A,E, 3A; Fig. S3C). It has been reported previously that long spindles can, eventually, form in *cik1Δ* cells, indicating that – with sufficient time – other activities can compensate for this loss (Gardner et al., 2008; Mieck et al., 2015). Our data suggest that Kar3Cik1 is necessary for maintaining proper spindle length during anaphase (Figs 2A, 3B). Vik1 had not previously been implicated in spindle length maintenance but we observed a statistically significant increase in spindle length during late anaphase in Vik1-depleted cells (Fig. 3B). It is possible that the increased length or number of cMTs in these cells (Fig. 3C) exerts a greater pulling force on SPBs to cause spindles to lengthen. This result might reflect a previously unknown function for Kar3Vik1. Another possibility is that, without available binding partners, monomeric Kar3 is affecting spindle and/or MT dynamics. This would have to be an indirect effect, as our data suggest that Kar3 does not bind MTs in the absence of accessory proteins and vice-versa (Fig. 2; Fig. S3). It could be that, in the absence of Kar3 heterodimers, MAPs that normally interact with these complexes are more abundant and able to affect MT dynamics. One example is Bim1, which only interacts with Kar3Cik1 heterodimers but not the separate monomers (Kornakov et al., 2020).

### Towards an integrated understanding of Kar3 function and regulation

Although much work has been done investigating Kar3 activity in the cell, most of this work focused on the Kar3Cik1 heterodimer. Here, we have systematically dissected the distinct localizations and functions of the two different Kar3 heterodimers during the cell cycle, and have uncovered previously unknown functions of Kar3Vik1. Our reconstituted MT dynamics assay implicates Kar3Vik1 as a modulator of MT dynamics and promoter of catastrophes. Localization data from our studies and those of others revealed that Kar3Vik1 is present exclusively in the cytoplasm, which implies that these activities affect only cMTs. Overall, Kar3Vik1 might primarily act to limit cMT length and number, particularly during anaphase – but perhaps during all of mitosis – to help control spindle length (Fig. 3B) and to, efficiently, orient the nucleus for high-fidelity chromosome inheritance, possibly by limiting the number of cMTs competent for pulling the daughter-bound SPB into the bud.

Across species, kinesin-14s serve a large variety of functions. The largest number of the kinesin-14s identified to date are found in land plants, which lack cytoplasmic dynein (Gicking et al., 2018). Presumably, these land plant kinesin-14s had to expand to fulfill all the roles normally performed by dynein in other organisms. Other examples of kinesin-14s include *Drosophila* Ncd (McDonald et al., 1990), mammalian HSET (Mountain et al., 1999), and fission yeast pkl1 and klp2 (Pidoux et al., 1996; Troxell et al., 2001). These kinesin-14s differ from budding yeast Kar3 in that they act as homodimers. Their function seems to be balancing forces within the spindle and capturing kinetochores, similar to Kar3Cik1. None of these systems have a corresponding kinesin-14 that exhibits activity exclusively on cytoplasmic or astral MTs. The complexity of Kar3 activities described here during the cell cycle suggests that budding yeast uses Kar3 for many of the same functions provided by kinesin-14s in other organisms. However, budding yeast has a closed mitosis and uses different kinesin-14 accessory proteins, i.e. Cik1 and Vik1, to specialize Kar3 function within the nucleus and the cytoplasm, respectively.

## MATERIALS AND METHODS

### Yeast strains, culturing and harvesting

All *Saccharomyces cerevisiae* strains in this study are of the s288c background and can be found in Table S1. C-terminal fluorescence and degradation tags were integrated into the genome by homologous recombination via lithium acetate transformation (Gietz and Woods, 2002). The *GFP-KAR3* allele was created by replacing a deletion cassette of *kar3Δ* in a diploid background with a P_KAR3_-EGFP-KAR3::KanMX fragment via lithium acetate transformation and sporulating to find GFP-positive haploids. Strains expressing *GFP-TUB1* and *mRUBY2-TUB1* were backcrossed into our lab strain at least four times to eliminate differences due to genetic background.

Except where noted, strains were grown in standard rich medium (YPD) at 25°C. Strains containing AID tag alleles were treated with a 1:2000 dilution of 0.5 M 3-indole acetic acid (Sigma-Aldrich, St. Louis, MO, USA) in 100% DMSO (250 µM and 0.05% final concentrations, respectively) and buffered with 50 mM potassium phosphate buffer at pH 6.2 for the 30 min prior to harvesting or imaging. Lysates were prepared with a SPEX cryogenic grinder (SPEX Sample Prep, Metuchen, NJ, USA) as described by Bergman et al. (2019).

### Live-cell imaging of yeast

Strains were grown overnight in rich medium at permissive temperature. They were then diluted to OD=0.1 in casamino acid medium (0.67% yeast nitrogen base with ammonium sulfate, 2% casamino acid, 2% glucose) supplemented with amino acids and grown at permissive temperature to early log phase. Cells were then shifted to 37°C for 3.5 h. If necessary, 3-IAA was added in the last 30 min of arrest to concentrations listed above. Cells were then mounted onto coverslips coated with concanavalin A (Sigma-Aldrich, St. Louis, MO, USA) and covered with pre-warmed medium. Microscopy was performed on a Nikon Eclipse Ti2 with a Nikon 60× Plan Apochromat objective and a 1.5× booster (Nikon, Tokyo, Japan) using an Orca Flash 4.0 camera (Hamamatsu, Shizuoka, Japan). Images were captured using Nikon Elements software. Temperature was maintained with a heated chamber and objective collar (Oko Labs, Pozzuoli, Italy). GFP was excited using a 488-laser line. mScarlet-I and mRUBY2 were excited using a 561-laser line. Eleven slice *z*-stack series spaced 0.5 µm apart were taken every 30 s for 10 min.

### Immunoblotting

Cleared lysates were prepared as described by Bergman et al. (2019). Lysate was diluted 1:9 in TURB sample buffer [62.5 mM Tris pH 6.8, 3 M Urea, 1% SDS, 0.05% (w/v) bromophenol blue, 5% β-mercaptoethanol]. Samples were then boiled for 5 min and analyzed by western blotting. Immunoblotting was performed with 1:750 9E10 mouse anti-Myc primary antibody (prepared from hybridoma supernatant), 1:1000 anti-GFP antibody (Roche, cat. #11814460001) and 1:40,000 anti-PGK antibody (Invitrogen, cat. #22C5D8). Band intensities were measured using Image Studio Lite (LI-COR, Lincoln, NE, USA).

### Glass slide cleaning and cover glass passivation

Glass slides were washed once for 30 min with acetone and then washed once for 30 min with 96% ethanol, and left to air dry. Cover glasses were passivated by sonication in acetone for 30 min, followed by a 15-min soak in 96% ethanol. The cover glasses were then rinsed three times with ddH_2_O and left in a 2% Hellmanex III (Hellma, Müllheim, Germany) solution for 2 h. Afterwards, the cover glasses were rinsed three times with ddH_2_O and dried with nitrogen gas. Cover glasses were then laid over a 50 µl drop of 100 mM HEPES, 1 mg ml^−1^ PLL-g-PEG and 1 mg ml^−1^ PLL-PEG-Biotin for 1 h in a humidity chamber. Cover glasses were then rinsed with 1× PBS for 2 min and then in ddH_2_O for 2 min. Finally, they were dried with nitrogen and stored at 4°C.

### Preparation for TIRF microscopy

Assembly of flow chambers for TIRF imaging was performed as described by Bergman et al. (2019). Similarly, generation of microtubule seeds followed according to Bergman et al. (2019), with the exception of using HiLyte 647-labeled tubulin (Cytoskeleton Inc., Denver, CO, USA) instead of rhodamine-labeled tubulin.

### Preparation of whole-cell lysates

For MT dynamics assays, lysates were prepared as described by Bergman et al. (2019), except that both exogenous ATP and GTP were excluded from the final clarified lysate as described by Torvi et al. (2022). Instead, 30 µl of clarified lysate was directly loaded into the flow chamber.

For characterization of GFP-Kar3 on microtubules *in vitro*, lysates were prepared as above with the following modifications. To enrich the population of soluble Kar3, frozen lysate powder was not diluted with 10× PEM buffer. Instead, 0.5 µl of Protease Inhibitor Cocktail IV was added to 0.24 g of lysate powder. The mixture was thawed on ice for 10 min and then sonicated for 2 s (1 s cycle time, 20% duty cycle, output control set to 2) using a W-385 sonicator with a #419 microtip (Heat Systems - Ultrasonics, Inc., Farmingdale, NY, USA) prior to ultracentrifugation.

### TIRF microscopy

MT dynamics reconstitution assays were performed on a Nikon Eclipse Ti2 inverted TIRF microscope using a Nikon 60× Plan Apochromat TIRF objective (NA 1.49) with a 1.5× booster lens (Nikon, Tokyo, Japan) in a temperature-controlled chamber (Oko Labs, Pozzuoli, Italy) warmed to 28°C and captured with an Orca Flash 4.0 camera (Hamamatsu, Shizuoka, Japan) operated by Nikon Elements software. Single-plane TIRF images of 488 nm and 561 nm illumination were taken every 5 s for 30 min.

Studies of motors on microtubules were performed on Nikon Eclipse Ti2 inverted microscope using a Nikon 60× CFI Apo TIRF objective (NA 1.49) and an Orca Fusion Gen III sCMOS camera (Hamamatsu, Shizuoka, Japan) at 1.5× magnification using the Nikon NIS Elements software. Using a LUNF 4-line laser launch (Nikon Instruments, Tokyo, Japan) and an iLas2 TIRF/FRAP module (Gataca Systems, Massy, France) total internal reflection fluorescence (TIRF) was performed to capture a single *z*-plane every 5 s for 20 min.

### Image and data analysis

Image files were analyzed using Fiji (NIH). Statistics were calculated using Graphpad Prism (Graphpad, San Diego, CA, USA). For finding an average spindle length, 50 spindles from either metaphase- or anaphase-arrested genotypes were counted. The average lengths of cytoplasmic MTs and the average number of cytoplasmic MTs per cell was calculated. This was done by counting and measuring the length and number of visible MTs outside the nucleus from 50 cells for each condition at the time-point zero. Statistical significance was determined by one-way ANOVA and using the Kruskal–Wallis test comparing the depleted strains to the WT. Fluorescence intensity along spindles was calculated by subtracting background fluorescence with a rolling ball average radius of 25 pixels, a sliding parabola and disabled smoothing. A 5-pixel wide line was used to measure SPB intensities from their center. Spindles were standardized to 100 points similar as described previously (Brownlee and Heald, 2019; Pedersen et al., 2020). Each point was then averaged and plotted with the standard deviation. For each condition 50 spindles were used, except for S phase of WT cells, for which only 40 were used. Fluorescence of Cik1-mScarlet-I and Vik1-mScarlet-I was analyzed in a similar manner, except that the window of observation was extended past the SPBs. Ten spindles of each strain were counted.

Kymographs for measuring dynamics were constructed from all MTs whose entire length was trackable for the entire 30-min observation window after registration. Dynamics parameters and statistics were calculated as described by Bergman et al. (2019).

## Supplementary Material

10.1242/joces.260621_sup1 Supplementary information
