# Supplementary material for: Cik1 and Vik1 accessory proteins confer distinct functions to the kinesin-14 Kar3
Source: J Cell Sci. 2023 Jun 13;136(11):jcs260621. doi: 10.1242/jcs.260621 (PMC10281266; doi:10.1242/jcs.260621)
Supplement: Supplementary information [file joces-136-260621-s1.pdf]

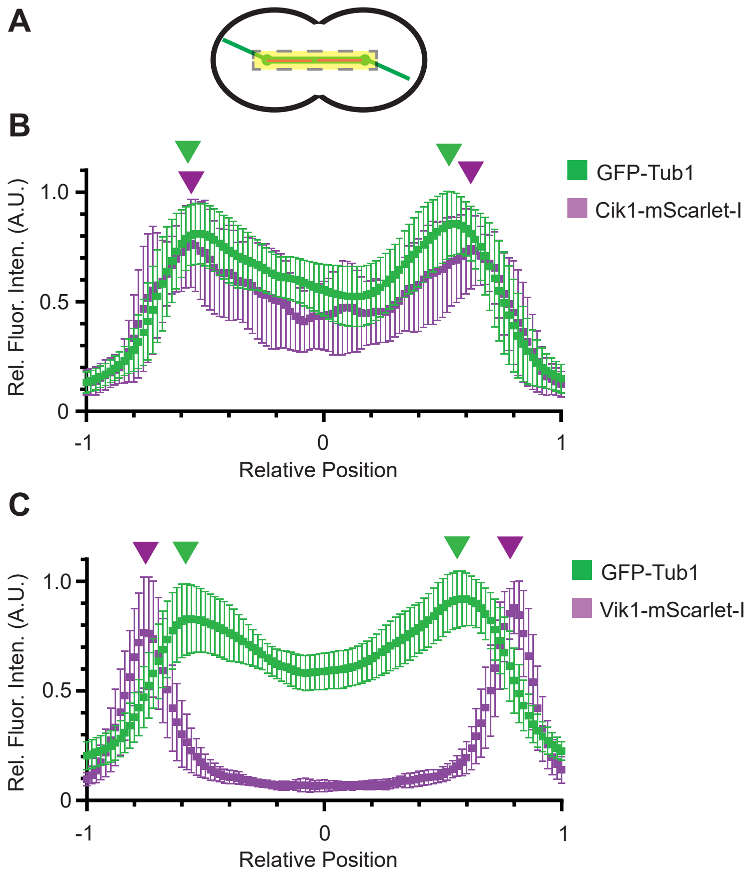

Fig. S1. Accessory Protein Localization During Metaphase. (A) Schematic of area measured for fluorescence intensity along spindles. Measurement of relative fluorescence intensities along the spindle from 10 metaphase-arrested cells expressing *GFP-TUB1* with either *CIK1-mScarlet-I* (**B**) or *VIK1-mScarlet-I* **(C)**. Straight lines were drawn extending past SPBs and the intensity of each channel was broken into 100 even parts as in Figure 1B. Max intensity from each channel for each trace was set to 1 The midpoint of the spindle set as 0 on the x-axis. Chevrons denote peak intensities for each channel on each end of the trace.

Journal of Cell Science • Supplementary information

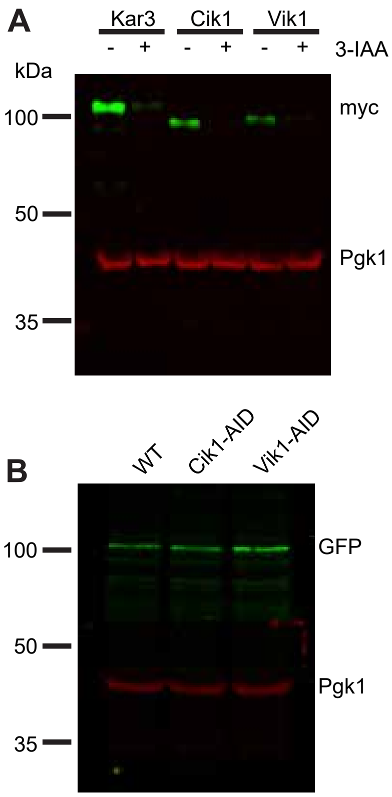

Fig. S2. Depletion of AID-tagged Proteins. (A) Strains expressing *GFP-TUB1 cdc23-1* and *KAR3-9myc-AID*, *CIK1-9myc-AID*, or *VIK1-9myc-AID* were prepared for lysates and either untreated or treated with 3-IAA for the 30 minutes prior to harvesting. Green bands are α-myc and red bands are α-Pgk1 as a loading control. Intensity measurements show that Kar3-AID and Cik1-AID are depleted by 90% and Vik1-AID is depleted by 75%. (B) Strains expressing *GFP-KAR3 cdc23-1* and either *CIK1-9myc-AID* or *VIK1-9myc-AID* were prepared for lysates and treated with 3-IAA for 30 minutes prior to harvesting. Green bands are α-GFP and red bands are α-PGK1 as a loading control. GFP-Kar3 levels are not decreased when either Cik1 or Vik1 is depleted.

Journal of Cell Science • Supplementary information

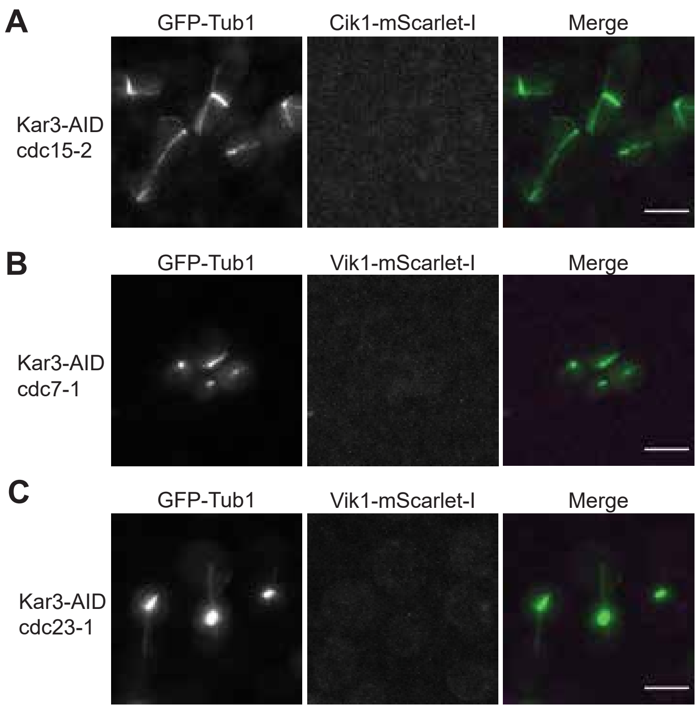

Fig. S3. Cik1 and Vik1 Localization Is Dependent Upon Kar3. *GFP-TUB1* (green) *KAR3-AID* cells were arrested and depleted of Kar3. **(A)** Cik1-mScarlet-I (magenta) in anaphase-arrested cells depleted of Kar3. **(B)** Vik1-mScarlet-I (magenta) localization in S phase without Kar3. **(C)** Vik1-mScarlet-I (magenta) localization in Kar3-depleted cells arrested in metaphase. Scale bars are 5 μm.

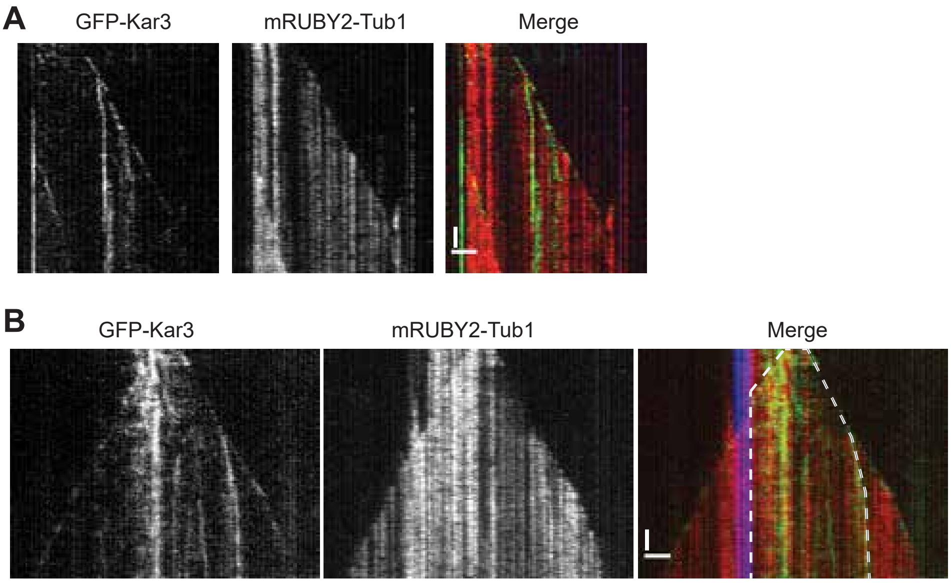

Fig. S4. Non-Motile Kar3 Diffusely Interacts with MTs in the Absence of Cik1 During Anaphase. Dynamic MTs were reconstituted from lysate arrested in anaphase and depleted of Cik1. GFP-Kar3 (green), mRUBY2-TUB1 (red), and HiLyte647-labeled seeds (blue). **(A)** GFP-Kar3 associated with a single MT. **(B)** GFP-Kar3 associated with an anti-parallel MT. Scale bars are 2 μm and vertical bars are 2 min Dashed lines surround the area of anti-parallel MT overlap.

Table S1. Strains Used in this Study

Strain Name	Genotype	Origin
DDY5662	*MATa, lys2-801, his3Δ-200, leu2-3, 112, ura3-52::GFP-TUB1::URA3, cdc28-4*	Bergman and Wong 2019
DDY5663	*MATa, lys2-801, his3Δ-200, leu2-3, 112, ura3-52::GFP-TUB1::URA3, cdc7-1*	Bergman and Wong 2019
DDY5664	*MATa, lys2-801, his3Δ-200, leu2-3, 112, ura3-52::GFP-TUB1::URA3, cdc23-1*	Bergman and Wong 2019
DDY5665	*MATa, lys2-801, his3Δ-200, leu2-3, 112, ura3-52::GFP-TUB1::URA3, cdc15-2*	Bergman and Wong 2019
DDY5674	*MATa, lys2-801, his3Δ-200, leu2-3, 112, KAR3-9myc-AID::HIS3, osTIR1::LEU2, ura3-52::GFP-TUB1::URA3, cdc28-4*	Bergman and Wong 2019
DDY5675	*MATa, lys2-801, his3Δ-200, leu2-3, 112, KAR3-9myc-AID::HIS3, osTIR1::LEU2, ura3-52::GFP-TUB1::URA3, cdc7-1*	Bergman and Wong 2019

Journal of Cell Science • Supplementary information

DDY5676	*MATa, lys2-801, his3Δ-200, leu2-3, 112, KAR3-9myc-AID::HIS3, osTIR1::LEU2, ura3-52::GFP-TUB1::URA3, cdc23-1*	Bergman and Wong 2019
DDY5677	*MATa, lys2-801, his3Δ-200, leu2-3, 112, KAR3-9myc-AID::HIS3, osTIR1::LEU2, ura3-52::GFP-TUB1::URA3, cdc15-2*	Bergman and Wong 2019
DDY5852	*MATa, lys2-801, his3Δ-200, leu2-3, 112, CIK1-9myc-AID::HIS3, osTIR1::LEU2, ura3-52::GFP-TUB1::URA3, cdc28-4*	This study
DDY5853	*MATa, lys2-801, his3Δ-200, leu2-3, 112, CIK1-9myc-AID::HIS3, osTIR1::LEU2, ura3-52::GFP-TUB1::URA3, cdc7-1*	This study
DDY5854	*MATa, lys2-801, his3Δ-200, leu2-3, 112, CIK1-9myc-AID::HIS3, osTIR1::LEU2, ura3-52::GFP-TUB1::URA3, cdc23-1*	This study
DDY5855	*MATa, lys2-801, his3Δ-200, leu2-3, 112, CIK1-9myc-AID::HIS3, osTIR1::LEU2, ura3-52::GFP-TUB1::URA3, cdc15-2*	This study

Journal of Cell Science • Supplementary information

DDY5856	*MATa, lys2-801, his3Δ-200, leu2-3, 112, VIK1-9myc-AID::HIS3, osTIR1::LEU2, ura3-52::GFP-TUB1::URA3, cdc28-4*	This study
DDY5857	*MATa, lys2-801, his3Δ-200, leu2-3, 112, VIK1-9myc-AID::HIS3, osTIR1::LEU2, ura3-52::GFP-TUB1::URA3, cdc7-1*	This study
DDY5858	*MATa, lys2-801, his3Δ-200, leu2-3, 112, VIK1-9myc-AID::HIS3, osTIR1::LEU2, ura3-52::GFP-TUB1::URA3, cdc23-1*	This study
DDY5859	*MATa, lys2-801, his3Δ-200, leu2-3, 112, VIK1-9myc-AID::HIS3, osTIR1::LEU2, ura3-52::GFP-TUB1::URA3, cdc15-2*	This study
DDY5860	*MATa, lys2-801, his3Δ-200, leu2-3, 112, TUB1::PHIS3-mRuby2-TUB1::HPH, EGFP-KAR3::KanMX, cdc28-4*	This study
DDY5861	*MATa, lys2-801, his3Δ-200, leu2-3, 112, TUB1::PHIS3-mRuby2-TUB1::HPH, EGFP-KAR3::KanMX, cdc7-1*	This study
DDY5862	*MATa, lys2-801, his3Δ-200, leu2-3, 112, TUB1::PHIS3-mRuby2-TUB1::HPH, EGFP-KAR3::KanMX, cdc23-1*	This study
DDY5863	*MATa, lys2-801, his3Δ-200, leu2-3, 112, TUB1::PHIS3-mRuby2-TUB1::HPH, EGFP-KAR3::KanMX, cdc15-2*	This study

Journal of Cell Science • Supplementary information

DDY5864	*MATa, lys2-801, his3Δ-200, leu2-3, 112, CIK1-9myc-AID::HIS3, osTIR1::LEU2, TUB1::PHIS3-mRuby2-TUB1::HPH, EGFP-KAR3::KanMX, cdc28-4*	This study
DDY5865	*MATa, lys2-801, his3Δ-200, leu2-3, 112, CIK1-9myc-AID::HIS3, osTIR1::LEU2, TUB1::PHIS3-mRuby2-TUB1::HPH, EGFP-KAR3::KanMX, cdc7-1*	This study
DDY5866	*MATa, lys2-801, his3Δ-200, leu2-3, 112, CIK1-9myc-AID::HIS3, osTIR1::LEU2, TUB1::PHIS3-mRuby2-TUB1::HPH, EGFP-KAR3::KanMX, cdc23-1*	This study
DDY5867	*MATa, lys2-801, his3Δ-200, leu2-3, 112, CIK1-9myc-AID::HIS3, osTIR1::LEU2, TUB1::PHIS3-mRuby2-TUB1::HPH, EGFP-KAR3::KanMX, cdc15-2*	This study
DDY5868	*MATa, lys2-801, his3Δ-200, leu2-3, 112, VIK1-9myc-AID::HIS3, osTIR1::LEU2, TUB1::PHIS3-mRuby2-TUB1::HPH, EGFP-KAR3::KanMX, cdc28-4*	This study
DDY5869	*MATa, lys2-801, his3Δ-200, leu2-3, 112, VIK1-9myc-AID::HIS3, osTIR1::LEU2, TUB1::PHIS3-mRuby2-TUB1::HPH, EGFP-KAR3::KanMX, cdc7-1*	This study

Journal of Cell Science • Supplementary information

DDY5870	*MATa, lys2-801, his3Δ-200, leu2-3, 112, VIK1-9myc-AID::HIS3, osTIR1::LEU2, TUB1::PHIS3-mRuby2-TUB1::HPH, EGFP-KAR3::KanMX, cdc23-1*	This study
DDY5871	*MATa, lys2-801, his3Δ-200, leu2-3, 112, VIK1-9myc-AID::HIS3, osTIR1::LEU2, TUB1::PHIS3-mRuby2-TUB1::HPH, EGFP-KAR3::KanMX, cdc15-2*	This study
DDY5872	*MATa, lys2-801, his3Δ-200, leu2-3, 112, CIK1-9myc-AID::HIS3, VIK1-9myc-AID::HIS3, osTIR1::LEU2, TUB1::PHIS3-mRuby2-TUB1::HPH, EGFP-KAR3::KanMX, cdc15-2*	This study
DDY5873	*MATa, lys2-801, his3Δ-200, leu2-3, 112, CIK1-mScarlet-I::HIS3, ura3-52::GFP-TUB1::URA3, cdc28-4*	This study
DDY5874	*MATa, lys2-801, his3Δ-200, leu2-3, 112, CIK1-mScarlet-I::HIS3, ura3-52::GFP-TUB1::URA3, cdc7-1*	This study
DDY5875	*MATa, lys2-801, his3Δ-200, leu2-3, 112, CIK1-mScarlet-I::HIS3, ura3-52::GFP-TUB1::URA3, cdc23-1*	This study
DDY5876	*MATa, lys2-801, his3Δ-200, leu2-3, 112, CIK1-mScarlet-I::HIS3, ura3-52::GFP-TUB1::URA3, cdc15-2*	This study
DDY5877	*MATa, lys2-801, his3Δ-200, leu2-3, 112, VIK1-mScarlet-I::HIS3, ura3-52::GFP-TUB1::URA3, cdc28-4*	This study

Journal of Cell Science • Supplementary information

DDY5878	*MATa, lys2-801, his3Δ-200, leu2-3, 112, VIK1-mScarlet-I::HIS3, ura3-52::GFP-TUB1::URA3, cdc7-1*	This study
DDY5879	*MATa, lys2-801, his3Δ-200, leu2-3, 112, VIK1-mScarlet-I::HIS3, ura3-52::GFP-TUB1::URA3, cdc23-1*	This study
DDY5880	*MATa, lys2-801, his3Δ-200, leu2-3, 112, VIK1-mScarlet-I::HIS3, ura3-52::GFP-TUB1::URA3, cdc15-2*	This study
DDY5881	*MATa, lys2-801, his3Δ-200, leu2-3, 112, KAR3-9myc-AID::HIS3, osTIR1::LEU2, CIK1-mScarlet-I::HIS3, ura3-52::GFP-TUB1::URA3, cdc15-2*	This study
DDY5882	*MATa, lys2-801, his3Δ-200, leu2-3, 112, KAR3-9myc-AID::HIS3, osTIR1::LEU2, VIK1-mScarlet-I::KanMX, ura3-52::GFP-TUB1::URA3, cdc7-1*	This study
DDY5883	*MATa, lys2-801, his3Δ-200, leu2-3, 112, KAR3-9myc-AID::HIS3, osTIR1::LEU2, VIK1-mScarlet-I::KanMX, ura3-52::GFP-TUB1::URA3, cdc23-1*	This study

Journal of Cell Science • Supplementary information
