# Supplementary material for: Cik1 and Vik1 accessory proteins confer distinct functions to the kinesin-14 Kar3
Source: J Cell Sci. 2023 Jun 13;136(11):jcs260621. doi: 10.1242/jcs.260621 (PMC10281266; doi:10.1242/jcs.260621)
Supplement: Supplementary file 1 [file joces-136-260621_review_history.pdf]

Cik1 and Vik1 accessory proteins confer distinct functions to the kinesin-14 Kar3

Zane J. Bergman, Jonathan J. Wong, David G. Drubin and Georjana Barnes
DOI: 10.1242/jcs.260621

Editor: Anne Straube

Review timeline

Original submission:	12 September 2022
Editorial decision:	10 November 2022
First revision received:	1 March 2023
Editorial decision:	4 April 2023
Second revision received:	30 April 2023
Accepted:	5 May 2023

Original submission

First decision letter

MS ID#: JOCES/2022/260621

MS TITLE: Cik1 and Vik1 Accessory Proteins Confer Distinct Functions to the Kinesin-14, Kar3

AUTHORS: Zane J. Bergman, Jonathan J. Wong, David G. Drubin, and Georjana Barnes
ARTICLE TYPE: Research Article

We have now reached a decision on the above manuscript.

To see the reviewers' reports and a copy of this decision letter, please go to: https://submit-jcs.biologists.org and click on the 'Manuscripts with Decisions' queue in the Author Area. (Corresponding author only has access to reviews.)

As you will see, the reviewers raise a number of substantial criticisms that prevent me from accepting the paper at this stage. They suggest, however, that a revised version might prove acceptable, if you can address their concerns. If you think that you can deal satisfactorily with the criticisms on revision, I would be pleased to see a revised manuscript. We would then return it to the reviewers.

Please ensure that you clearly highlight all changes made in the revised manuscript. Please avoid using 'Tracked changes' in Word files as these are lost in PDF conversion.

I should be grateful if you would also provide a point-by-point response detailing how you have dealt with the points raised by the reviewers in the 'Response to Reviewers' box. Please attend to all of the reviewers' comments. If you do not agree with any of their criticisms or suggestions please explain clearly why this is so.

© 2023. Published by The Company of Biologists under the terms of the Creative Commons Attribution License (https://creativecommons.org/licenses/by/4.0/).

Reviewer 1

Advance summary and potential significance to field

Kar3 is an interesting motor because it is known to pair with one of two different non-catalytic partners, Cik1 or Vik1. This study by Bergman and colleagues seeks to understand how Cik1 or Vik1 promote different programs of Kar3 subcellular localization and activity. The authors use the budding yeast system to combine fluorescent fusion proteins expressed from their native loci, conditional depletion alleles of each subunit to establish dependencies, and TIRF imaging of cell extracts to measure activity without the constraints of cellular compartmentalization. The major conclusions are that Cik1 and Vik1 promote Kar3 localization to distinct microtubule structures and at different points in the cell cycle, and that Kar3-Cik1 vs Kar3-Vik1 complexes exhibit unique activities in vitro.

Although the study makes good use of the extract system developed previously by the authors, there are major flaws related to impact and rigor. Kar3 is perhaps the best-studied yeast kinesin, and the roles of Cik1 and Vik1 in directing its localization to nucleus or cytoplasm, and its function are relatively well established. The current study revisits these questions with some new tools, but the conclusions do not advance our understanding beyond previous models. The depletion alleles and cell cycle-related results here hint at molecular-level regulation of Kar3, but the study does not pursue the mechanistic contributions of Cik1 and Vik1, certainly not as far as previous work by the Westermann and Gardner labs. The weaknesses in rigor are related to statements that that are not supported by the data presented in the figures and comparisons that are not clearly supported by statistical analyses. These issues weaken support for the conclusions. These issues are detailed below.

Comments for the author

Major concerns

1) The imaging data presented in Figures 1 and 2 do not support the statements in the results. Here are several examples:

Page 6. “Cells arrested in G1 had the stereotypical bright nuclear microtubule bundle and long cytoplasmic MTs (cMTs) with a single GFP-Kar3 spot at the spindle pole body (SPB).” The images in Figure 1A do not show an obvious spot of GFP-Kar3 signal.

Page 6. “For cells arrested in metaphase, short spindles were fully covered in GFP-Kar3.” I do not see an obvious difference between GFP-Kar3 localization in S-phase vs M-phase. Both show strong signal at the poles and weaker signal on presumably nuclear microtubules. What is the definition of ‘fully covered’?

Page 6. “We also observed, at very low frequency, GFP spots on the plus ends of cMTs.” These spots are not clear in the images.

Page 7. “Vik1 localized to SPBs (Fig. 1C), indicating that the single spot seen at the SPB in G1-arrested GFP-Kar3 cells consisted of only Kar3Vik1 heterodimers.” Similar to Kar3, these spots are not visible in the images.

Page 7. “Vik1-mScarlet-I was found mainly on the cytoplasmic side of SPBs”. The images shown here do not clearly show the cytoplasmic side of the SPB (vs nuclear side on inner plaque, or on short microtubules near the SPB). The microscopy used here is not suited to support this claim. This same comment applies to all other descriptions of the cytoplasmic or nuclear face of the SPB.

There are more examples like these. In general, these claims should either be re-written to more accurately describe the presented data, or the authors should include new images that are more representative. In addition, the study would benefit by quantifying the localization of Kar3, Cik1 or Vik1. This would be particularly helpful for demonstrating consistency across cells and replicates and for comparing localization across cell cycle stages and identifying significant changes.

© 2023. Published by The Company of Biologists under the terms of the Creative Commons Attribution License (https://creativecommons.org/licenses/by/4.0/).

2) Several of the findings suggest that Kar3-Cik1 and Kar3-Vik1 exhibit different levels of activity during different cell cycle stages. Can any of these differences in activity be explained by changes in protein levels? This question is important to address, particularly since Figure 1 shows little to no localization for Kar3 or Cik1 during G1, or Vik1 during G1 or S phase. The authors could address this by using western blots of cells arrested at G1, S M and Anaphase, similar to the imaging experiments.

3) Are the appropriate statistical analyses used in Figure 4? Assuming that the bars shown in these plots are mean and SD (I could not find this information), many of these distributions show a great degree of overlap, yet are reported as significantly different from one another (<0.05). Are these p-values from t-tests comparing means?

4) Figure 5E and F. These results are difficult to interpret. The figure legend and methods do not describe how many microtubules were compared, and in how many experiments, and there do not appear to be any statistical analyses. What can we conclude from these data?

5) The reconstitution experiments using cell extracts are limited to measuring Kar3 localization and plus end regulation on single microtubules and do not capture activity on multiple microtubules. Multiple studies have described roles for Kar3-Cik1 and Kar3-Vik1 on parallel microtubules. Therefore the extract experiments may not be ideal for measuring Kar3-Cik1 or Kar3-Vik1 on its native microtubule substrate. This should be addressed in the discussion.

6) The AID alleles are not validated by data shown in the figures. For example the statement on page 8 “Western blot analysis showed that this was sufficient time for these proteins to be depleted by over 90%” is not supported by data shown in the manuscript. This data is important for interpreting the results and should be included.

Minor concerns

1) Figure 2. Labeling each of these panels would help clarify which protein(s) is depleted.

2) It’s surprising that the authors did not extend their study to mating cells. Kar3-Cik1 is known to regulate microtubules in mating cells, so compare activity in cells or cell extracts during mating could help provide a complete picture of Kar3 regulation.

3) Introduction, page 3:

“The MAPs responsible for this regulation are either completely excluded from one compartment, or are post-translationally modified differentially across compartments, to give rise to these different functions in the nucleus and in the cytoplasm.”

This statement is overly broad. There are many MAPs that associate with both nuclear and cytoplasmic microtubules. If the authors are referring to specific examples of differential post-translational modification, they should include citations to direct readers to the relevant literature.

4) Page 7 “…with Cik1 transporting the kinesin into the nucleus via its nuclear-localization sequence and Vik1 retaining it in the cytoplasm.” What is the evidence that Cik1 transports Kar3 into the nucleus? Alternatively, Kar3 could itself be transported into the nucleus, and its binding to Cik1 in the nucleus could promote nuclear retention. The authors should omit this statement if their data do not clearly support it.

5) Page 13. “MTs in Cik1-depleted lysates grew 85.6% of the time and only paused 1.2% of the time.” This does not accurately describe the chart in Figure 4C.

© 2023. Published by The Company of Biologists under the terms of the Creative Commons Attribution License (https://creativecommons.org/licenses/by/4.0/).

Reviewer 2

Advance summary and potential significance to field

The authors study how the localization and activity of the budding yeast kinesin-14 Kar3 depend on its binding partners Cik1 and Vik1. This extends previous work of the authors and other labs in the field. Notable here is the systematic approach and the high quality of the experiments and the careful quantification of microtubule dynamics data. In several experiments accute loss of function after auxin-induced degradation is studied. Microtubule dynamics are measured in yeast extract, an innovative assay that has recently been developed by the authors. This study goes beyond their previous work by performing various cell cycle arrests to measure the activity of Kar3/Cik1 and Kar3/Vik1 at different cell cycle stages, adding more detailed knowledge about the functional differences between these two heterodimers. This study is in part confirmatory and in part provides a valuable resource for the field. Although in general the manuscript is well written, it is not always easy to grasp the conceptual novelty that can be derived from the various careful quantifications, given the fairly descriptive nature of the presentation. Said that, the experiments appear to have been performed to a technically very high standard, making this study a useful and likely reliable resource.

Comments for the author

1. The main concern is probably that the yeast extract may represent a mixed nucleo-cytoplasmic extract and given that the two heterodimers localize and function in different compartments (nucleus vs cytoplasm), the question arises how representative the measured effects, for example on microtubule dynamics in the different cell cycle states are for the situation in nucleus and cytosplasm respectively. This point may deserve a discussion.

2. A technical question: is Kar3 of the heterodimer also degraded when its partner Cik1 or Vik1 is degraded (and vice versa)? It would be useful to state what is known about this (including evidence if there is any).

3. The manuscript may be more accessible to the non-expert if the authors could state more explicitly when presenting the various results (e.g. the many dynamic instability parameters), to which extent the observations are novel, similar to or different from previous measurements, and to which extent they support or contradict what's currently known about the function of the two heterodimeric complexes. At least to this reader, the specific motivation for the various measurements was not always clear. The discussion helps, but it would be a more interesting read if some more context could be spelled out earlier.

Reviewer 3

Advance summary and potential significance to field

This manuscript from Bergman et al. examines the roles of the kinesin accessory proteins, Cik1 and Vik1, in directing Kar3 localization and influencing Kar3 functions on microtubules. The general localization of Kar3 dependence on the accessory factors has been previously reported, along with the known segregation of Cik1 and Vik1 functions to the nucleus and cytoplasm, respectively. This study goes further by taking a clever approach and arresting cells in specific stages of the cell cycle followed by depletion of Kar3, either subunit, or both. This can remove non-specific secondary effects that may occur in previous cell cycles and/or stages. The authors also use an approach they developed previously to reconstitute dynamic yeast microtubules in whole cell extracts. They apply this to examine microtubule behavior in extracts lacking Kar3, Cik1, or Vik1 prepared from cells in each of the cell cycle stages. The authors also use this approach to examine Kar3 end-tracking and motility properties in the extracts. This novel approach reveals that the influence of Kar3 on microtubule dynamics differs at various cell stages, and that Cik1 and Vik1 may exert unique influences on Kar3 function.

© 2023. Published by The Company of Biologists under the terms of the Creative Commons Attribution License (https://creativecommons.org/licenses/by/4.0/).

Comments for the author

While this study has the potential to generate an impactful manuscript, the current version is far from being suitable for publication. Overall, this manuscript contains multiple instances of inaccurate descriptions of data or comparisons, apparent issues with image cropping/scaling, fluorescence image intensity profiles, and a consistent deficiency with proper use of citations. Despite making many claims regarding relative localization of Kar3, Cik1, or Vik1, there is a dearth of quantitative analysis of localization in this manuscript, which leaves most related conclusions unsupported. The comments below highlight many examples, but are likely not an exhaustive list.

This manuscript needs extensive work before it can be considered for publication. Based on the current version, the student/postdoc and senior author/mentor should work together to ensure the student/postdoc understands the appropriate use and function of citations, and to think more deeply about the relationship between these data and how they support the conclusions, including the limitations, as well as potential alternatives that are also consistent with the reported data.

Example Issues:
- There is a general lack of proper citation throughout the article. For example, near the end of the first paragraph in the introduction there are several statements made that would benefit from citation. For instance, MAPs are completely excluded from compartments or PTMs are different across compartments.

- The writing and citations are also loose in many places. For example, on line 86 “While these studies were pioneering efforts to elucidate Kar3 function, they might have suffered from complicating issues such as use of heterogeneous assay constituents and innovative but nevertheless non-physiological recombinant fusion proteins to stably form each of the two Kar3 heterodimers (Chu et al., 2005; Rank et al., 90 2012)” The citations here are confusing. The sentence is referring to previously mentioned and cited pioneering studies, but Chu and Rank were not described previously. There is no explanation of whether Chu and Rank suffered from certain issues, nor how these studies relate to the previous work.

- The authors cite Maddox et al. 2003 in a group citation simply stating that fluorescently tagged Kar3 has been previously used to ascertain localization. What they fail to address is that Kar3-GFP has been previously observed to localize to the plus-ends of cMTs in G1 arrest, and that localization is coupled to MT dynamics. In the current study, GFP-Kar3 displays no such localization in G1 arrested cells. Moreover, Vik1 localization is uncoupled from Kar3 localization in these cells in this study. This issue is simply not addressed. This seems egregious considering the authors propose that differences in constructs may contribute to confusing results among previous studies.

- Equally important, do the authors have any evidence that their tagged constructs function properly in these various cell cycle states, e.g. shmooing/mating, etc.?

- Line 144: The authors do not observe Vik1 on the plus-ends of cMTs, yet they do observe GFP-Kar3. Although the authors state this, they do not address the issue at all. This is interesting as they propose that differences in constructs may contribute to confusing results among previous studies. In this current study, Localization of Cik1 or Vik1, or of Kar3 are used to infer concomitant localization of the corresponding partner. However, here is a case where they are not observed in a consistent manner. Do the authors have any information, current or previously published, to evaluate the functionality or behavior of their chosen constructs?

- Line 140, “while Vik1 was observed on the spindle poles at SPBs, presumably on the cytoplasmic SPB face.” The next two sentences say, “Upon arrest in metaphase, …

Vik1-mScarlet-I was found mainly on the cytoplasmic side of SPBs”. In two sentences this goes from presumably on the cytoplasmic face based on other reports, which are speculated by these authors to possibly have complicating issues, to definitively on the cytoplasmic face. Actually, there is no attempt to determine this in the current manuscript.

- Line 170: “During metaphase in cells depleted of Cik1, GFP-Kar3 was absent from the nucleus, but a strong GFP-Kar3 signal was observed on the cytoplasmic face of the SPB.” The current evidence

© 2023. Published by The Company of Biologists under the terms of the Creative Commons Attribution License (https://creativecommons.org/licenses/by/4.0/).

presented does not discriminate between the inner and outer SPB face. Was there any quantitative analysis to determine whether the wide-field signal is really further apart than the mRUBY-Tub1 signal? Is this interpretation based on previously published analyses of Cik1 function?

- Line 180: "In anaphase-arrested cells depleted of Vik1, GFP-Kar3 was absent from the cytoplasm but present on the nuclear face of SPBs with a cloud of signal near them, consistent with kinetochore localization." Similarly to the cytoplasmic SPB face, without quantitative analysis how are the authors concluding wide-field signal is on the nuclear vs. cytoplasmic face of the SPB?

- Line 363: "This observation matches with our in vivo data showing that GFP-Kar3 localizes with both sides of the SPB, from which yeast MTs. GFP-Kar3 no longer localized to the SPB on the nuclear or cytoplasmic side of the nuclear envelope when the corresponding Kar3 accessory protein was depleted." It is not possible to make this claim without verification of signal to either face of the SPB along with quantification on either side.

- Line 487: "These data, combined with analysis of strains expressing a degron-tagged version of an accessory protein and GFP-Kar3, independently confirmed that Kar3Cik1 resides on the nuclear face of SPBs," This conclusion cannot be drawn without having analysis of GFP-Kar3, or Cik1 localization relative to the SPB, e.g. perhaps an SPB protein. The wide-field data shown is consistent with either the nuclear or cytoplasmic face.

- It appears that there is a significant error with the image scaling, intensity and/or error bars. For example, in Fig 1 the error bars are 5 microns. The size and number of cells that fit in each frame are highly inconsistent. Same with the relative cMT or spindles in various frames. The cells arrested in G1, S, M and A look appear very differently sized, as do their microtubule components.

- There also appears to be inconsistencies with scaling of image intensities. For instance, in Fig 2A and 2B G1 cells, and in 2C +IAA the Kar3 signal appears to be cumulatively much more intense than in the other panels. Is this the case? If so, then why? Alternatively, is the software displaying the intensities on a dynamic scale so that the very low, diffuse signal appears brighter because it is more consistent with background? This is seriously compounded by the lack of quantitative analysis.

- Line 275: "4C). MTs in Cik1- depleted lysates grew 85.6% of the time and only paused 1.2% of the time." This manuscript makes several statements about how the lysate approach contains PTMs and other factors to represent the cellular state. But the authors seem to ignore the cases in which differences are evident, which may be revealing additional regulation of Kar3 in vivo. For instance, if MTs grow 85% of the time in G1 Cik1-AID lysates, and also grow faster but shorten at the same rate, and even more impressive have just 10% of catastrophes, would this predict that the in vivo cMT length in Cik1-AID would be very much longer than in Kar3-AID cells? The difference in length in vivo is not so remarkable.

- In some places there is either minimal consideration of how well a conclusion is supported by the data, or appears to be perhaps no consideration. As just some examples, but not exhaustive, leading up to line 229, Kar3 depleted cells have anaphase spindle lengths similar to WT, but either Cik1 or Vik1 depleted cells have longer spindles. "Since these two accessory proteins are in different cellular compartments and the magnitude of this increase is different, the mechanism of increasing the length of spindles most likely differs." There are more possibilities, for instance, the lack of either accessory protein may result in excess Kar3 that, without a partner, contributes to excessive spindle elongation.

- The paragraph from lines 299-316 for metaphase-arrested cells presents and describes data for Cik1 and Vik1 depleted extracts that is essentially similar in both cases throughout the paragraph. However, the paragraph inexplicitly ends with the conclusion "These data suggest that, during metaphase, Kar3Cik1 and Kar3Vik1 regulate MT dynamics by separate mechanisms." Frankly, there is no way to imagine how this conclusion is drawn, or even partially supported by the preceding data.

- On line 366: These observations are followed by the conclusion: "We observed minus-end directed motility of Kar3 on 11.1% of MTs in lysates from WT cells. Motility was not observed in the Cik1-depleted lysates. Only 1.9% of the MTs in Vik1-depleted lysates had motors moving in the minus-end

© 2023. Published by The Company of Biologists under the terms of the Creative Commons Attribution License (https://creativecommons.org/licenses/by/4.0/).

direction. These data suggest that the majority of minus-end-directed motility we observed in our assay is dependent upon Kar3Cik1.” This conclusion is not accurate. There is 100% loss of minus-end movements in Cik1-depleted, and there is 83% loss in Vik1-depletion. The movements are nearly equally dependent on both Cik1 and Vik1, and you may need both present for the vast majority of these movements. (might these be more than single Kar3 heterodimers?)

- The paragraph on anaphase (lines 374-381) does not contain any values or quantitative comparisons. This is in contrast to all the sections prior. It makes it harder to appreciate the significance of these differences in relation to the other data. How important might the speculated role for Vik1 be in anaphase? What should this statement be based on?

- On line 409 is a particularly clear example of a failure to use citations. The sentence states, “We found that though these heterodimers are very similar in structure, they…” Nowhere in this manuscript is data presented on the structures of Vik1 and Cik1, or their heterodimers. Nowhere in this manuscript are the structures of Cik1 or Vik1 or their heterodimers discussed nor cited previously. This is essentially a random statement not supported by data or citation(s).

- Line 514: Another example. “Mutations in KAR3 and CIK1 both lead to shorter metaphase spindles that collapse or break as mitosis progresses.” This is not data presented in the current manuscript, and it has no citation to support the statement.

- Line 613: “For MT dynamics assays, lysates were prepared as in (Bergman et al., 2019) except both exogenous ATP and GTP were excluded from the final clarified lysate. Instead, 30 μL of clarified lysate was directly loaded into the flow chamber.” Can the authors provide a rationale for why they excluded both ATP and GTP from these preparations whereas they included them before? This appears potentially significant. Is it know whether this may affect the apparent influences of Kar3/Cik1 or Kar3/Vik1 in combination with all the other regulators present in the lysate?

- The authors state that diffuse Kar3 particles coat antiparallel microtubules. There does not appear to be any quantification of this. In Fig S2 A there are few motile particles. In B there are also few. What is the relative percentage? In B, it is hard to decern the antiparallel section. In fact, only one seed is visible. Can the authors mark the antiparallel section? How many of these motile and non-motile Kar3 particles are within the antiparallel zone? How are they determined to be coated compared to other microtubules? (The figure title has typos.)

- Line 123: “When cells were arrested in S phase, the majority of cells had short spindles and long cMTs.” How is long being defined? In nearly all the cells shown there are no cMTs visible. In Fig 3 it appears S phase cells have the shortest cMTs relative to the other stages.

- The authors may wish to examine their choice of representative images and/or the phenotypes of S-arrested cells with Vik1- and Cik1-mScarlet labels. Compared to GFP-Kar3 cells, which have short bipolar spindles, the other two cell types seem to possess both uni- and very short bi-polar spindles.

- Line 151: “These observations reaffirm that Kar3 localization changes in a programmed manner throughout the vegetative cell cycle and is dependent upon its binding partner, with Cik1 transporting the kinesin into the nucleus via its nuclear-localization sequence and Vik1 retaining it in the cytoplasm.” The qualitative evidence presented does not seem to fully support this claim for Vik1. It appears that Vik1-associated Kar3 does not change localization in a cell cycle dependent manner. Rather, it is at the SPB with a slight ‘haze’ in M phase. Neither is seen at cMT plus-ends.

- Line 162: “Western blot analysis showed that this was sufficient time for these proteins to be depleted by over 90%.” Is this data missing or is this data not shown?

- Line 167: “Cells arrested in G1 and depleted of Cik1 retained a single spot of GFP-Kar3 at SPBs as was seen in non-depleted cells (Fig. 2A).” There is no spot of GFP-Kar3 at the SPB visible in any G1 cells in Fig. 2A, nor in non-depleted cells in Fig. 1A. Is this statement accurate? Are incorrect cells displayed? Can the intensity relative to cytoplasmic signal be quantified?

- Line 172: “The increase in GFP-Kar3 on cMTs could be due to more Vik1 binding to Kar3 in the absence of Cik1.” There is no Kar3 visible on cMTs in these cells. Is this statement referring to the

© 2023. Published by The Company of Biologists under the terms of the Creative Commons Attribution License (https://creativecommons.org/licenses/by/4.0/).

signal that is at the SPB? If so, it is uncertain whether it is on ‘cMTs’ and needs to be clarified. If not, it is inaccurate.

- Line 178: “GFP-Kar3 had a reduced signal at the SPBs in S phase-arrested cells 179 depleted of Vik1.” Is this statement compared to Cik1compared to Cik1-AID S-phase cells or non-AID control cells? More importantly, quantitative analysis of localization seems crucial to make claims such as this example.

- Line 188: “When these cells were arrested and the Kar3 binding partners depleted, as above, no GFP-Kar3 could be detected at the SPBs or in association with the nucleus (Fig 2C).” The authors should select another field of cells for +IAA. It is understandable that spindles may collapse under these conditions, but it is impossible to discern which buds and mothers go together. More concerning, 4 of the 6 mothers or buds have spindle signal. Thus, they appear not to be large budded metaphase arrested cells.

- Line 238: “Interestingly, the lengths of cMTs in Kar3- and Vik1-depleted cells were almost indistinguishable from WT during all cell cycle stages except G1, where all depletions increased the number of cMTs (Figs.3D-F).” This statement seems partially inaccurate and needs more explanation. cMTs in Kar3-depeleted cells are significantly longer in metaphase (Fig. 3F) and similar for Vik1-depleted cells in Anaphase.

- Line 273: “These MTs also disassembled faster (0.74 ± 0.36 and 0.97 ± 0.35 μm min-1, 274 respectively).” This statement is incorrect. The rates are being compared to Kar3-AID lysates with 0.78 rate.

- Line 350: “This observation matches our in vivo data (Fig. 2B) wherein GFP-Kar3 does not have strong SPB signal in S phase arrested, Vik1-depleted cells.” This is difficult to conclude, especially without any quantitative analyses. What is the signal being compared to, cells with both Cik1 and Vik1 dependent signal at the SPB? How much is contributed from each? And does it matter that the comparison includes what the authors state includes both the outer and inner SPB face in one case?

- Line 431: “When the gene encoding Cik1 or Vik1 is deleted, the catastrophe frequency decreases and the overall dynamicity of MTs increases because assembly and disassembly rates increase.” Are the authors referring to this study? If not, a reference is needed. If so, it appears the genes were not deleted, nor expression reduced. Instead, a degron system was used on the protein product.

- Line 433: “Interestingly, the Kar3Cik1 heterodimer appears to control the number of catastrophes during G1, whereas Kar3Vik1 has a more prominent role in S phase.” This is another example of when the authors overlook caveats of their in vitro system. The authors make claims about relative Cik1 and Vik1 activities, yet do not account for the fact that they, and other components are segregated in vivo, but homogeneously mixed in the in vitro lysates. Might other factors that are segregated in vivo also contribute to have an effect on either protein? This should at least be considered.

- Line 502: “Consistently, SPB staining in cells was only slightly diminished in Cik1-depleted cells, but was nearly absent in the Vik1-depleted cells.” This manuscript makes several conclusions about the amount and localization of proteins, yet there is no quantitative analyses to support these statements/claims. This is even more concerning because the scales and displayed intensity ranges of images appears inconsistent among the various strains and conditions.

- Line 506: “We also observed an increase in cMT numbers for Kar3- and Vik1-depleted cells at all cell cycle stages (Fig. 3C).” Can the authors include any statistical comparisons of these numbers? This may help readers to interpret the strength of these differences because in some cases they appear fairly similar.

- Fig 5A, B, D: Arrows should be included to show the indicated molecule.

- Fig. 5E: Y-axis reports fraction and it seems the text reports percentage. It would aid the reader to be consistent in both places. The same may be true for Fig. 5F. Additionally, these panels may not be cited properly within the text.

© 2023. Published by The Company of Biologists under the terms of the Creative Commons Attribution License (https://creativecommons.org/licenses/by/4.0/).

- On line 385: “In our assay, GFP-Kar3 moves toward the minus end at a rate of 0.64 ± 0.27 μm min-1 (10.6 nm min-1) in metaphase-arrested lysate” How is this calculated? It seems like there must be a typo.

- Line 535: “Overall, Kar3Vik1 may primarily act to limit cMT length and numbers, particularly during anaphase (but perhaps all of mitosis) to help control spindle length and to help efficiently orient the nucleus for efficient chromosome inheritance.” This statement would benefit from a little more clarity on how the authors think limiting cMT length and numbers will control spindle length. More importantly, is there evidence, in this study or others, that limiting cMT length and numbers would help efficiently orient the nucleus for efficient chromosome inheritance? If so, it should be presented/cited. If not, the speculation should be made clear and supported.

- Line 310: “compared to MTs in WT lysates (Figure 3I).” Should read (Figure 4I).

- Line 314: “in that the frequency of catastrophe and dynamics were increased” Should this read “catastrophe and dynamicity’?

- Line 217: “The above observations are consistent with those in previous reports on spindles in kar3Δ, cik1Δ, and vik1Δ cells (Hepperla et al., 2014), which indicate that the Kar3Cik1 motor is needed to both balance forces along the interpolar MTs and to capture kinetochores during metaphase.” The last part of this sentence, to capture kinetochores during metaphase, cannot be concluded from a difference in spindle length. More explanation is needed to support this statement.

- Line 552: “Having separate Kar3populations with different accessory factors allows budding yeast to compartmentalize microtubule functions and cell cycle regulation.” This sentence is not clear. Having a closed mitosis allows yeast to compartmentalize, even if they use the same heterodimer in both. Different Kar3 accessory proteins then utilize that compartmentalization.

- Line 559: “Fluorescent and degradation tags were integrated into the genome by homologous recombination as previously described.” Described where? How?

- Line 261: “Strains containing AID tag alleles were treated with final concentrations of 250 μM 3-indole acetic acid (Sigma-Aldrich, St. Louis, MO, USA) in DMSO and buffered with 50 mM potassium phosphate buffer at pH 6.2 for the 30 minutes prior to harvesting or imaging.” Regarding DMSO, how much was in the experimental media? What is important is the final concentration of DMSO. This can also be deduced from stating the stock or final concentration if it were provided.

- Line 569: “Strains were grown overnight in rich media at permissive temperature. They were then diluted to OD = 0.1 in casamino acid media (0.67% yeast nitrogen base with ammonium sulfate, 2% casamino acid, 2% glucose) supplemented with amino acids and grown at permissive temperature to recover.” It is unclear what is meant by ‘to recover’. They are growing in rich media at permissive temperature. What are they recovering from?

- Line 599: Is Hellmanex III common knowledge? It seems like this may need a citation or source.

First revision

Author response to reviewers' comments

Dear Dr. Straube,

We thank the reviewers and you for your critical reading of our manuscript. We appreciate the comments, concerns, and suggestions given and have tried to address them to improve the paper. Our responses to the individual comments are presented below in green. All changes made to the

© 2023. Published by The Company of Biologists under the terms of the Creative Commons Attribution License (https://creativecommons.org/licenses/by/4.0/).

manuscript itself are highlighted in yellow in the revised manuscript. We thank you and the reviewers for the constructive comments, which have helped us to make substantial improvements to the manuscript. We hope that the corrections made and clarifications of our methods and reasoning are sufficient to merit approval for publication, and that our article can be included in the *JCS* special issue on Cell Biology of Motors.

Sincerely,
Zane Bergman, Jonathan Wong, David Drubin and Georjana Barnes

Reviewer 1 Comments for the Author:
Major concerns

1) The imaging data presented in Figures 1 and 2 do not support the statements in the results. Here are several examples:

Page 6. “Cells arrested in G1 had the stereotypical bright nuclear microtubule bundle and long cytoplasmic MTs (cMTs) with a single GFP-Kar3 spot at the spindle pole body (SPB).” The images in Figure 1A do not show an obvious spot of GFP-Kar3 signal.

We have replaced original images with new, improved images that we hope more clearly support that statements we make in the Results. Arrowheads were added to direct readers’ attention to details.

Page 6. “For cells arrested in metaphase, short spindles were fully covered in GFP-Kar3.” I do not see an obvious difference between GFP-Kar3 localization in S- phase vs M-phase. Both show strong signal at the poles and weaker signal on presumably nuclear microtubules. What is the definition of ‘fully covered’?

“...localized along the length of the mitotic spindle”

To address this important point, we quantified the fluorescence intensity of GFP-Kar3 along spindles and added a panel to Figures 1 and 2. These new plots quantitatively show the differences in intensity at either different stages of the cell cycle or under different conditions. The text was modified to highlight these differences.

Page 6. “We also observed, at very low frequency, GFP spots on the plus ends of cMTs.” These spots are not clear in the images.

Along with later comments, the reviewers pointed out that there was insufficient data presented to confirm the localization of either GFP-Kar3 or Vik1-mScarlet-I to the plus- ends of MTs. We have therefore removed any reference to this conclusion from the manuscript as we were not able to gather enough data to fully support this localization.

Page 7. “Vik1 localized to SPBs (Fig. 1C), indicating that the single spot seen at the SPB in G1-arrested GFP-Kar3 cells consisted of only Kar3Vik1 heterodimers.” Similar to Kar3, these spots are not visible in the images.

Refer to above comment; mention of this localization result has been removed.

Page 7. “Vik1-mScarlet-I was found mainly on the cytoplasmic side of SPBs”. The images shown here do not clearly show the cytoplasmic side of the SPB (vs nuclear side on inner plaque, or on short microtubules near the SPB). The microscopy used here is not suited to support this claim. This same comment applies to all other descriptions of the cytoplasmic or nuclear face of the SPB.

There are more examples like these. In general, these claims should either be re- written to more accurately describe the presented data, or the authors should include new images that are more representative. In addition, the study would benefit by quantifying the localization of Kar3, Cik1 or Vik1. This would be particularly helpful for demonstrating consistency across cells and replicates, and for comparing localization across cell cycle

© 2023. Published by The Company of Biologists under the terms of the Creative Commons Attribution License (https://creativecommons.org/licenses/by/4.0/).

stages and identifying significant changes.

In order to better document the relative localization of Cik1 or Vik1 to SPBs, we have now included line scans of the fluorescence intensity of either Cik1-mScarlet-I or Vik1- mScarlet-I compared to GFP-tubulin. The peak of GFP fluorescence was taken as the center of the SPB, which is embedded in the nuclear membrane. Our scans show that the majority of the Cik1-mScarlet fluorescence occurs at and between the SPB peaks of a metaphase spindle, whereas the Vik1-mScarlet fluorescence is outside of these peaks. Along with other evidence from previous work, we believe the conclusions that Kar3Cik1 is in the nucleus and Kar3Vik1 is in the cytoplasm are well supported. We have included the average of 10 scans as Fig. S1 to support our claim.

2) Several of the findings suggest that Kar3-Cik1 and Kar3-Vik1 exhibit different levels of activity during different cell cycle stages. Can any of these differences in activity be explained by changes in protein levels? This question is important to address, particularly since Figure 1 shows little to no localization for Kar3 or Cik1 during G1, or Vik1 during G1 or S phase. The authors could address this by using western blots of cells arrested at G1, S M and Anaphase, similar to the imaging experiments.

Although we agree that these data would be informative, we believe that they are beyond the scope of this study. Several proteome-wide surveys have indicated wildly different numbers of each of these molecules depending on the method used (see yeastgenome.org for references). Our new images provide localizations for these proteins during the corresponding cell cycle stages, but generating decisive quantitative assessments of the different proteins' levels as a function of the cell cycle stage would be a significant undertaking.

3) Are the appropriate statistical analyses used in Figure 4? Assuming that the bars shown in these plots are mean and SD (I could not find this information), many of these distributions show a great degree of overlap, yet are reported as significantly different from one another (<0.05). Are these p-values from t-tests comparing means?

For the data presented in Figure 4, we used the student's pair-wise t test to determine significance between data sets as we did in our previous paper. We have updated the figure legend to include both the meaning of the bars (mean with SD) and the statistical analysis used. The differences are indeed statistically significant.

4) Figure 5E and F. These results are difficult to interpret. The figure legend and methods do not describe how many microtubules were compared, and in how many experiments, and there do not appear to be any statistical analyses. What can we conclude from these data?

We agree with the reviewer that these data are difficult to interpret due to the small number of events that occurred during our observations. The total number of MTs that we observed, and the events, were reported in Table 2. We have added this information to the figure legend to direct readers there. Additionally, we have added SEM to the bar graphs to show the spread of the data we are reporting.

5) The reconstitution experiments using cell extracts are limited to measuring Kar3 localization and plus end regulation on single microtubules and do not capture activity on multiple microtubules. Multiple studies have described roles for Kar3-Cik1 and Kar3-Vik1 on parallel microtubules. Therefore, the extract experiments may not be ideal for measuring Kar3-Cik1 or Kar3-Vik1 on its native microtubule substrate. This should be addressed in the discussion.

We thank the reviewer for making this important point. We now emphasize in our discussion that our reconstitution studies focused on single MTs, which is an advantage, but one that carries the indicated caveat. While it is true that Kar3 heterodimers, in particular Kar3Cik1, have been shown to function on parallel MTs, this does not mean that these motors do not have effects on single MTs. Most previous in vitro studies have ascribed function to Kar3 based on single MT studies, and papers from the Tanaka lab have shown that Kar3 functions on single MTs to recapture kinetochores.

6) The AID alleles are not validated by data shown in the figures. For example, the statement on page 8 "Western blot analysis showed that this was sufficient time for these proteins to be

© 2023. Published by The Company of Biologists under the terms of the Creative Commons Attribution License (https://creativecommons.org/licenses/by/4.0/).

depleted by over 90%" is not supported by data shown in the manuscript. This data is important for interpreting the results and should be included.

We agree with the reviewers and have addressed this point by including a western blot of lysates from cell cultures either untreated or treated with 3-IAA as Figure S2.

Minor concerns

1) Figure 2. Labeling each of these panels would help clarify which protein(s) is depleted.

Labels have been added to the figures.

2) It's surprising that the authors did not extend their study to mating cells.
Kar3-Cik1 is known to regulate microtubules in mating cells, so compare activity in cells or cell extracts during mating could help provide a complete picture of Kar3 regulation.

Though it is true that Kar3Cik1 takes on new roles in mating cells, and that other data suggests that Kar3Vik1 is downregulated in this life cycle stage, we believe that such an analysis is beyond the scope of our paper, which focuses on the vegetative life cycle.
We agree that the proposed experiments could be part of a very interesting future study.

3) Introduction, page 3:

"The MAPs responsible for this regulation are either completely excluded from one compartment, or are post-translationally modified differentially across compartments, to give rise to these different functions in the nucleus and in the cytoplasm."

This statement is overly broad. There are many MAPs that associate with both nuclear and cytoplasmic microtubules. If the authors are referring to specific examples of differential post-translational modification, they should include citations to direct readers to the relevant literature.

These statements have been amended to include more specific examples to help clarify our points.

4) Page 7 "…with Cik1 transporting the kinesin into the nucleus via its nuclear- localization sequence and Vik1 retaining it in the cytoplasm." What is the evidence that Cik1 transports Kar3 into the nucleus? Alternatively, Kar3 could itself be transported into the nucleus, and its binding to Cik1 in the nucleus could promote nuclear retention. The authors should omit this statement if their data do not clearly support it.

The reviewer is correct in that a mechanism for Kar3's localization into the nucleus has not been elucidated. We have omitted this statement so as to not mislead readers.

5) Page 13. "MTs in Cik1-depleted lysates grew 85.6% of the time and only paused 1.2% of the time." This does not accurately describe the chart in Figure 4C.

Good catch! We have fixed this error in the manuscript.

Reviewer 2 Comments for the Author:
1. The main concern is probably that the yeast extract may represent a mixed nucleo-cytoplasmic extract and given that the two heterodimers localize and function in different compartments (nucleus vs cytoplasm), the question arises how representative the measured effects, for example on microtubule dynamics in the different cell cycle states are for the situation in nucleus and cytosplasm, respectively. This point may deserve a discussion.

This is a fair point and we have therefore increased emphasis of this point in our revised Discussion. . Despite this caveat, our ability to view activities on single microtubules, to control the cell cycle stage, and to genetically knockdown specific components of the system, plus our complementary, validating in vivo analyses, lead us to believe that our observations are unique and valuable contributions.

© 2023. Published by The Company of Biologists under the terms of the Creative Commons Attribution License (https://creativecommons.org/licenses/by/4.0/).

2. A technical question: is Kar3 of the heterodimer also degraded when its partner Cik1 or Vik1 is degraded (and vice versa)? It would be useful to state what is known about this (including evidence if there is any).

Neither we nor other labs have reported on this possibility. We did not perform a western blot analysis of protein levels of non-targets of our depletion conditions. Our live-cell images show that the background fluorescence from GFP-Kar3 increases in strains that are depleted of either Cik1 or Vik1, suggests either that Kar3 is not degraded when the accessory protein levels are depleted, or that GFP is cleaved off of Kar3 and remains intact.

3. The manuscript may be more accessible to the non-expert if the authors could state more explicitly when presenting the various results (e.g. the many dynamic instability parameters), to which extent the observations are novel, similar to or different from previous measurements, and to which extent they support or contradict what's currently known about the function of the two heterodimeric complexes. At least to this reader, the specific motivation for the various measurements was not always clear. The discussion helps, but it would be a more interesting read if some more context could be spelled out earlier.

This is an excellent point. We have taken this matter into consideration when revising the manuscript and have added context to guide readers through the findings, particularly for Figure 4.

Reviewer 3 Comments for the Author:
- There is a general lack of proper citation throughout the article. For example, near the end of the first paragraph in the introduction there are several statements made that would benefit from citation. For instance, MAPs are completely excluded from compartments or PTMs are different across compartments.

See my comments for Reviewer 1.

- The writing and citations are also loose in many places. For example, on line 86 “While these studies were pioneering efforts to elucidate Kar3 function, they might have suffered from complicating issues such as use of heterogeneous assay constituents and innovative but nevertheless non-physiological recombinant fusion proteins to stably form each of the two Kar3 heterodimers (Chu et al., 2005; Rank et al., 90 2012)” The citations here are confusing. The sentence is referring to previously mentioned and cited pioneering studies, but Chu and Rank were not described previously. There is no explanation of whether Chu and Rank suffered from certain issues, nor how these studies relate to the previous work.

We regret this mistake in the placement of this citation and have removed it from this sentence.

- The authors cite Maddox et al. 2003 in a group citation simply stating that fluorescently tagged Kar3 has been previously used to ascertain localization. What they fail to address is that Kar3-GFP has been previously observed to localize to the plus-ends of cMTs in G1 arrest, and that localization is coupled to MT dynamics. In the current study, GFP-Kar3 displays no such localization in G1 arrested cells. Moreover, Vik1 localization is uncoupled from Kar3 localization in these cells in this study. This issue is simply not addressed. This seems egregious considering the authors propose that differences in constructs may contribute to confusing results among previous studies.

As mentioned above, we have removed mention of GFP-Kar3 or Vik1-mScarlet localization to MT plus-ends due to a lack of sufficient supporting data. However, it should be noted that the studies this reviewer is referencing show Kar3-GFP on cMT plus-ends in mating cells, not vegetative cells in G1. These are two very different life- cycle stages with different MT functions.

- Equally important, do the authors have any evidence that their tagged constructs function properly in these various cell cycle states, e.g. shmooing/mating, etc.?

© 2023. Published by The Company of Biologists under the terms of the Creative Commons Attribution License (https://creativecommons.org/licenses/by/4.0/).

Observation of cell growth rates, spindle morphology and function and of differences in MT dynamics in auxin untreated and treated lysates, we believe that function is preserved in the tagged constructs. Not all of the data supporting this conclusion were included in the manuscript. Also, in our previous paper, strains containing the *KAR3-AID* alleles used in this study were paired with *KIP3* deletions. Disruptions in *KIP3* and *KAR3* genes are synthetically lethal. The tagged alleles did not show synthetic lethality of any genetic interaction, and so they maintain function.

- Line 144: The authors do not observe Vik1 on the plus-ends of cMTs, yet they do observe GFP-Kar3. Although the authors state this, they do not address the issue at all. This is interesting as they propose that differences in constructs may contribute to confusing results among previous studies. In this current study, Localization of Cik1 or Vik1, or of Kar3 are used to infer concomitant localization of the corresponding partner. However, here is a case where they are not observed in a consistent manner. Do the authors have any information, current or previously published, to evaluate the functionality or behavior of their chosen constructs?

As mentioned above, we have removed mention of GFP-Kar3 or Vik1-mScarlet localization to MT plus-ends due to insufficient supporting data.

- Line 140, “while Vik1 was observed on the spindle poles at SPBs, presumably on the cytoplasmic SPB face.” The next two sentences say, “Upon arrest in metaphase, … Vik1-mScarlet-I was found mainly on the cytoplasmic side of SPBs”. In two sentences this goes from presumably on the cytoplasmic face based on other reports, which are speculated by these authors to possibly have complicating issues, to definitively on the cytoplasmic face. Actually, there is no attempt to determine this in the current manuscript.

We agree that this wording is confusing and have amended this passage. Additionally, quantification of fluorescent proteins along spindles was conducted to help clarify their position relative to SPBs (see comments above). These data have been included in Figures 1, 2, and S1 to help place the Kar3 heterodimers in cellular compartments.

- Line 170: “During metaphase in cells depleted of Cik1, GFP-Kar3 was absent from the nucleus, but a strong GFP-Kar3 signal was observed on the cytoplasmic face of the SPB.” The current evidence presented does not discriminate between the inner and outer SPB face. Was there any quantitative analysis to determine whether the wide-field signal is really further apart than the mRUBY-Tub1 signal? Is this interpretation based on previously published analyses of Cik1 function?

See above.

- Line 180: “In anaphase-arrested cells depleted of Vik1, GFP-Kar3 was absent from the cytoplasm but present on the nuclear face of SPBs with a cloud of signal near them, consistent with kinetochore localization.” Similarly to the cytoplasmic SPB face, without quantitative analysis how are the authors concluding wide-field signal is on the nuclear vs. cytoplasmic face of the SPB?

See above.

- Line 363: “This observation matches with our in vivo data showing that GFP-Kar3 localizes with both sides of the SPB, from which yeast MTs. GFP-Kar3 no longer localized to the SPB on the nuclear or cytoplasmic side of the nuclear envelope when the corresponding Kar3 accessory protein was depleted.” It is not possible to make this claim without verification of signal to either face of the SPB along with quantification on either side.

See above.

- Line 487: “These data, combined with analysis of strains expressing a degron-tagged version of an accessory protein and GFP-Kar3, independently confirmed that Kar3Cik1 resides on the nuclear face of SPBs,” This conclusion cannot be drawn without having analysis of GFP-Kar3, or Cik1 localization relative to the SPB, e.g. perhaps an SPB protein. The wide-field data shown is consistent with either the nuclear or cytoplasmic face.

See above.

© 2023. Published by The Company of Biologists under the terms of the Creative Commons Attribution License (https://creativecommons.org/licenses/by/4.0/).

- It appears that there is a significant error with the image scaling, intensity and/or error bars. For example, in Fig 1 the error bars are 5 microns. The size and number of cells that fit in each frame are highly inconsistent. Same with the relative cMT or spindles in various frames. The cells arrested in G1, S, M and A look appear very differently sized, as do their microtubule components.

We have addressed these issues with new images. The different cell cycle arrests leave cells at different sizes. The *cdc28-4* allele in particular causes defects in growth polarity. Since we are not pre-synchronizing cells before arrest, cells throughout the population enter the arrest at different times and may increase in size dramatically if they arrest quickly due to their cell cycle position at the time of the temperature shift.

- There also appears to be inconsistencies with scaling of image intensities. For instance, in Fig 2A and 2B G1 cells, and in 2C +IAA the Kar3 signal appears to be cumulatively much more intense than in the other panels. Is this the case? If so, then why? Alternatively, is the software displaying the intensities on a dynamic scale so that the very low, diffuse signal appears brighter because it is more consistent with background? This is seriously compounded by the lack of quantitative analysis.

When making new images, we subtracted the background. We hope this satisfactorily addresses this issue. Another consideration, especially for 2C, is that without accessory proteins, there is more GFP-Kar3 in the background/cytoplasm and not on MTs.

- Line 275: "4C). MTs in Cik1- depleted lysates grew 85.6% of the time and only paused 1.2% of the time." This manuscript makes several statements about how the lysate approach contains PTMs and other factors to represent the cellular state. But the authors seem to ignore the cases in which differences are evident, which may be revealing additional regulation of Kar3 in vivo. For instance, if MTs grow 85% of the time in G1 Cik1-AID lysates, and also grow faster but shorten at the same rate, and even more impressive have just 10% of catastrophes, would this predict that the in vivo cMT length in Cik1-AID would be very much longer than in Kar3-AID cells? The difference in length in vivo is not so remarkable.

The reviewer raises an interesting point in that despite increased rates and duration of MT growth, particularly in the case of lysates from Cik1-AID cells in G1, this does not translate into a much larger difference in cMT lengths in live cells. The average cMT length in G1-arrested cells is ~2.8 um and this average increases to 4.3 um when Cik1 is depleted. Therefore, cMTs are 1.5X longer when Cik1 is absent. This is not so unremarkable in cells that are roughly 6-7 um long. We also agree with the reviewer that there may be more to MT length regulation in vivo than is revealed in our assays. Chief among many possibilities is spatial constraints. In our previous paper, reviewers pointed out that this important component was missing in our reconstitution assay, and we made note of it in the Discussion of that paper. Regulation of MTs by spatial constraints and cues is an interesting topic for future studies.

- In some places there is either minimal consideration of how well a conclusion is supported by the data, or appears to be perhaps no consideration. As just some examples, but not exhaustive, leading up to line 229, Kar3 depleted cells have anaphase spindle lengths similar to WT, but either Cik1 or Vik1 depleted cells have longer spindles. "Since these two accessory proteins are in different cellular compartments and the magnitude of this increase is different, the mechanism of increasing the length of spindles most likely differs." There are more possibilities, for instance, the lack of either accessory protein may result in excess Kar3 that, without a partner, contributes to excessive spindle elongation.

The *GFP-KAR3 CIK1-AID VIK1-AID cdc23-1* live cell imaging data show that without either accessory protein, GFP-Kar3 does not associate with MTs. How this effect impacts spindle elongation would probably depend on its ability to bind other MAPs or regulatory proteins (kinases/phosphatases). However, Stefan Westermann's lab has shown that Kar3 does not bind Bim1 without being in complex with Cik1. We now discuss these considerations in the revised manuscript.

© 2023. Published by The Company of Biologists under the terms of the Creative Commons Attribution License (https://creativecommons.org/licenses/by/4.0/).

- The paragraph from lines 299-316 for metaphase-arrested cells presents and describes data for Cik1 and Vik1 depleted extracts that is essentially similar in both cases throughout the paragraph. However, the paragraph inexplicitly ends with the conclusion “These data suggest that, during metaphase, Kar3Cik1 and Kar3Vik1 regulate MT dynamics by separate mechanisms.” Frankly, there is no way to imagine how this conclusion is drawn, or even partially supported by the preceding data.

We agree that this closing statement is contradictory to the data presented in the paragraph. It has been rewritten to reflect that these activities are similar and that there is a synergistic effect of removing both activities in our assay.

- On line 366: These observations are followed by the conclusion: “We observed minus- end directed motility of Kar3 on 11.1% of MTs in lysates from WT cells. Motility was not observed in the Cik1- depleted lysates. Only 1.9% of the MTs in Vik1-depleted lysates had motors moving in the minus-end direction. These data suggest that the majority of minus-end-directed motility we observed in our assay is dependent upon Kar3Cik1.” This conclusion is not accurate. There is 100% loss of minus-end movements in Cik1- depleted, and there is 83% loss in Vik1-depletion. The movements are nearly equally dependent on both Cik1 and Vik1, and you may need both present for the vast majority of these movements. (might these be more than single Kar3 heterodimers?)

The reviewer is correct. Movement of GFP-Kar3 along single MTs in our reconstitution assay seems to require both heterodimers to be present in metaphase. We have corrected the relevant manuscript text. We agree that the observations might indicate that there are “more than single Kar3 heterodimers”.

- The paragraph on anaphase (lines 374-381) does not contain any values or quantitative comparisons. This is in contrast to all the sections prior. It makes it harder to appreciate the significance of these differences in relation to the other data. How important might the speculated role for Vik1 be in anaphase? What should this statement be based on?

We have corrected this oversight by adding values into the text in this paragraph. We agree with the reviewer that this makes a better case for why Vik1 is important in anaphase.

- On line 409 is a particularly clear example of a failure to use citations. The sentence states, “We found that though these heterodimers are very similar in structure, they…” Nowhere in this manuscript is data presented on the structures of Vik1 and Cik1, or their heterodimers. Nowhere in this manuscript are the structures of Cik1 or Vik1 or their heterodimers discussed nor cited previously. This is essentially a random statement not supported by data or citation(s).

The reviewer is correct in that crystal structures of native Kar3Cik1 heterodimers have not been solved and the way we have written this passage suggests that they have been. We have revised this passage to say that structures predicted from domains and studies on truncated proteins suggest that Kar3Cik1 and Kar3Vik1 have structural similarity at a gross level but do have important distinctions.

- Line 514: Another example. “Mutations in KAR3 and CIK1 both lead to shorter metaphase spindles that collapse or break as mitosis progresses.” This is not data presented in the current manuscript, and it has no citation to support the statement.

These data are part of Fig 3A and is in the text describing these data.

- Line 613: “For MT dynamics assays, lysates were prepared as in (Bergman et al., 2019) except both exogenous ATP and GTP were excluded from the final clarified lysate. Instead, 30 μL of clarified lysate was directly loaded into the flow chamber.” Can the authors provide a rationale for why they excluded both ATP and GTP from these preparations whereas they included them before? This appears potentially significant. Is it know whether this may affect the apparent influences of Kar3/Cik1 or Kar3/Vik1 in combination with all the other regulators present in the lysate?

We updated our assay to exclude exogenous ATP and GTP as we determined that a sufficient amount of these nucleotides exists in the lysate to maintain the system throughout our

© 2023. Published by The Company of Biologists under the terms of the Creative Commons Attribution License (https://creativecommons.org/licenses/by/4.0/).

observation time window based on motility of Kip3 even in the presence of hexokinase (Torvi et al. 2022) and polymerization of MTs. This exclusion did not significantly change MT dynamics from one trial to the next. We believe that by not adding these nucleotides we remove a small dilution of the lysates and make them more reflective of what is happening in cells. These considerations are now mentioned in the Materials and Methods section.

- The authors state that diffuse Kar3 particles coat antiparallel microtubules. There does not appear to be any quantification of this. In Fig S2 A there are few motile particles. In B there are also few. What is the relative percentage? In B, it is hard to decern the antiparallel section. In fact, only one seed is visible. Can the authors mark the antiparallel section? How many of these motile and non-motile Kar3 particles are within the antiparallel zone? How are they determined to be coated compared to other microtubules? (The figure title has typos.)

As this was a passing observation that agreed with previous studies, we put these data into a supplementary figure that was not quantified. We thank the reviewer for pointing out that our presentation was difficult to interpret, so we added graphics to aid readers in understanding the data presented, and we corrected the title. Further work on these types of interactions would be interesting, but beyond the scope of this study.

- Line 123: “When cells were arrested in S phase, the majority of cells had short spindles and long cMTs.” How is long being defined? In nearly all the cells shown there are no cMTs visible. In Fig 3 it appears S phase cells have the shortest cMTs relative to the other stages.

We thank the reviewer for pointing out this discrepancy between our data and text. The text has been corrected to accurately reflect that cMTs in our *cdc7-1* arrested cells are the shortest we observed.

- The authors may wish to examine their choice of representative images and/or the phenotypes of S-arrested cells with Vik1- and Cik1-mScarlet labels. Compared to GFP- Kar3 cells, which have short bipolar spindles, the other two cell types seem to possess both uni- and very short bi-polar spindles.

We have replaced the images presented to reflect the majority of cells that we observed.

- Line 151: “These observations reaffirm that Kar3 localization changes in a programmed manner throughout the vegetative cell cycle and is dependent upon its binding partner, with Cik1 transporting the kinesin into the nucleus via its nuclear- localization sequence and Vik1 retaining it in the cytoplasm.” The qualitative evidence presented does not seem to fully support this claim for Vik1. It appears that Vik1- associated Kar3 does not change localization in a cell cycle dependent manner. Rather, it is at the SPB with a slight ‘haze’ in M phase. Neither is seen at cMT plus-ends.

This passage has been rewritten with the reviewer’s suggestions taken into account. It is true that Vik1 does not change localization throughout the cell cycle, and we have removed any text referencing Kar3Vik1 at cMT plus ends.

- Line 162: “Western blot analysis showed that this was sufficient time for these proteins to be depleted by over 90%.” Is this data missing or is this data not shown?

We have added a supplementary figure (S2) to show the depletion of these proteins in our lysates and updated the text to inform readers of the extent of depletion.

- Line 167: “Cells arrested in G1 and depleted of Cik1 retained a single spot of GFP- Kar3 at SPBs as was seen in non-depleted cells (Fig. 2A).” There is no spot of GFP- Kar3 at the SPB visible in any G1 cells in Fig. 2A, nor in non-depleted cells in Fig. 1A. Is this statement accurate? Are incorrect cells displayed? Can the intensity relative to cytoplasmic signal be quantified?

We have replaced the images and have added arrowheads to assist readers in finding key features.

© 2023. Published by The Company of Biologists under the terms of the Creative Commons Attribution License (https://creativecommons.org/licenses/by/4.0/).

- Line 172: “The increase in GFP-Kar3 on cMTs could be due to more Vik1 binding to Kar3 in the absence of Cik1.” There is no Kar3 visible on cMTs in these cells. Is this statement referring to the signal that is at the SPB? If so, it is uncertain whether it is on ‘cMTs’ and needs to be clarified. If not, it is inaccurate.

See above comments about Kar3Vik1 on cMT plus-ends.

- Line 178: “GFP-Kar3 had a reduced signal at the SPBs in S phase-arrested cells 179 depleted of Vik1.” Is this statement compared to Cik1compared to Cik1-AID S- phase cells or non-AID control cells? More importantly, quantitative analysis of localization seems crucial to make claims such as this example.

We have changed the text to clarify this statement. Additionally, GFP-Kar3 fluorescence intensity was quantified and added to Fig 1.

- Line 188: “When these cells were arrested and the Kar3 binding partners depleted, as above, no GFP-Kar3 could be detected at the SPBs or in association with the nucleus (Fig 2C).” The authors should select another field of cells for +IAA. It is understandable that spindles may collapse under these conditions, but it is impossible to discern which buds and mothers go together. More concerning, 4 of the 6 mothers or buds have spindle signal. Thus, they appear not to be large budded metaphase arrested cells.

We have processed these images so that it is easier to distinguish the morphology of the cells and their microtubule structures.

- Line 238: “Interestingly, the lengths of cMTs in Kar3- and Vik1-depleted cells were almost indistinguishable from WT during all cell cycle stages except G1, where all depletions increased the number of cMTs (Figs.3D-F).” This statement seems partially inaccurate and needs more explanation. cMTs in Kar3-depeleted cells are significantly longer in metaphase (Fig. 3F) and similar for Vik1-depleted cells in Anaphase.

We have amended this statement to make it clearer for readers, and have amended the paragraph to more thoroughly describe the other conditions in which cMT lengths are longer than WT.

- Line 273: “These MTs also disassembled faster (0.74 ± 0.36 and 0.97 ± 0.35 µm min-1, 274 respectively).” This statement is incorrect. The rates are being compared to Kar3- AID lysates with 0.78 rate.

We thank the reviewer for finding this mistake. We have corrected it.

- Line 350: “This observation matches our in vivo data (Fig. 2B) wherein GFP-Kar3 does not have strong SPB signal in S phase arrested, Vik1-depleted cells.” This is difficult to conclude, especially without any quantitative analyses. What is the signal being compared to, cells with both Cik1 and Vik1 dependent signal at the SPB? How much is contributed from each? And does it matter that the comparison includes what the authors state includes both the outer and inner SPB face in one case?

We have added language to this passage to indicate that we are comparing GFP-Kar3 fluorescence levels in S phase cells that are either WT or depleted of Cik1 or Vik1.

- Line 431: “When the gene encoding Cik1 or Vik1 is deleted, the catastrophe frequency decreases and the overall dynamicity of MTs increases because assembly and disassembly rates increase.” Are the authors referring to this study? If not, a reference is needed. If so, it appears the genes were not deleted, nor expression reduced. Instead, a degron system was used on the protein product.

The reviewer is right that these data are from this study and are protein depletions. The text was corrected.

- Line 433: “Interestingly, the Kar3Cik1 heterodimer appears to control the number of catastrophes during G1, whereas Kar3Vik1 has a more prominent role in S phase.” This is another example of when the authors overlook caveats of their in vitro system. The authors make claims

© 2023. Published by The Company of Biologists under the terms of the Creative Commons Attribution License (https://creativecommons.org/licenses/by/4.0/).

about relative Cik1 and Vik1 activities, yet do not account for the fact that they, and other components are segregated in vivo, but homogeneously mixed in the in vitro lysates. Might other factors that are segregated in vivo also contribute to have an effect on either protein? This should at least be considered.

- Line 502: “Consistently, SPB staining in cells was only slightly diminished in Cik1- depleted cells, but was nearly absent in the Vik1-depleted cells.” This manuscript makes several conclusions about the amount and localization of proteins, yet there is no quantitative analyses to support these statements/claims. This is even more concerning because the scales and displayed intensity ranges of images appears inconsistent among the various strains and conditions.

We have included quantitation of GFP-Kar3, Cik1-mScarlet, and Vik1-mScarlet along spindles in our updated manuscript and have updated all of our cell images. We believe that these changes address the issues raised in this comment.

- Line 506: “We also observed an increase in cMT numbers for Kar3- and Vik1-depleted cells at all cell cycle stages (Fig. 3C).” Can the authors include any statistical comparisons of these numbers? This may help readers to interpret the strength of these differences because in some cases they appear fairly similar.

We have updated the data presented in Fig 3C to include error and statistical significance. We have updated the text to reflect the data analysis. We thank the reviewer for this suggestion.

- Fig 5A, B, D: Arrows should be included to show the indicated molecule.

We have added arrowheads to these figures to assist readers.

- Fig. 5E: Y-axis reports fraction and it seems the text reports percentage. It would aid the reader to be consistent in both places. The same may be true for Fig. 5F. Additionally, these panels may not be cited properly within the text.

We have changed axes in figures and figure legend to “Percentage” to stay consistent with the text.

- On line 385: “In our assay, GFP-Kar3 moves toward the minus end at a rate of 0.64 ± 0.27 μm min-1 (10.6 nm min-1) in metaphase-arrested lysate” How is this calculated? It seems like there must be a typo.

We thank the reviewer for pointing out this error and we have corrected it.

- Line 535: “Overall, Kar3Vik1 may primarily act to limit cMT length and numbers, particularly during anaphase (but perhaps all of mitosis) to help control spindle length and to help efficiently orient the nucleus for efficient chromosome inheritance.” This statement would benefit from a little more clarity on how the authors think limiting cMT length and numbers will control spindle length. More importantly, is there evidence, in this study or others, that limiting cMT length and numbers would help efficiently orient the nucleus for efficient chromosome inheritance? If so, it should be presented/cited. If not, the speculation should be made clear and supported.

The reviewer correctly points out that this part of the Discussion is conjecture without the authors stating so. We now include language to inform readers that this is a proposed mechanism.

- Line 310: “compared to MTs in WT lysates (Figure 3I).” Should read (Figure 4I).

This has been fixed in the text.

- Line 314: “in that the frequency of catastrophe and dynamics were increased” Should this read “catastrophe and dynamicity’?

This has been fixed in the text.

© 2023. Published by The Company of Biologists under the terms of the Creative Commons Attribution License (https://creativecommons.org/licenses/by/4.0/).

- Line 217: "The above observations are consistent with those in previous reports on spindles in kar3Δ, cik1Δ, and vik1Δ cells (Hepperla et al., 2014), which indicate that the Kar3Cik1 motor is needed to both balance forces along the interpolar MTs and to capture kinetochores during metaphase." The last part of this sentence, to capture kinetochores during metaphase, cannot be concluded from a difference in spindle length. More explanation is needed to support this statement.

We have removed the last part of this statement.

- Line 552: "Having separate Kar3populations with different accessory factors allows budding yeast to compartmentalize microtubule functions and cell cycle regulation." This sentence is not clear. Having a closed mitosis allows yeast to compartmentalize, even if they use the same heterodimer in both. Different Kar3 accessory proteins then utilize that compartmentalization.

We have removed this statement to enhance clarity and remove ambiguity.

- Line 559: "Fluorescent and degradation tags were integrated into the genome by homologous recombination as previously described." Described where? How?

We have updated this section of the Materials and Methods to assist readers.

- Line 261: "Strains containing AID tag alleles were treated with final concentrations of 250 µM 3-indole acetic acid (Sigma-Aldrich, St. Louis, MO, USA) in DMSO and buffered with 50 mM potassium phosphate buffer at pH 6.2 for the 30 minutes prior to harvesting or imaging." Regarding DMSO, how much was in the experimental media? What is important is the final concentration of DMSO. This can also be deduced from stating the stock or final concentration if it were provided.

3-IAA was made at 0.5 M concentration in 100% DMSO. This solution was then diluted 1:2000 when added to cultures for final concentrations of 250 µM 3-IAA and 0.05% DMSO. These details were added this to Materials and Methods.

- Line 569: "Strains were grown overnight in rich media at permissive temperature. They were then diluted to OD = 0.1 in casamino acid media (0.67% yeast nitrogen base with ammonium sulfate, 2% casamino acid, 2% glucose) supplemented with amino acids and grown at permissive temperature to recover." It is unclear what is meant by 'to recover'. They are growing in rich media at permissive temperature. What are they recovering from?

Changed "to recover" to "to early log phase" to clarify our method.

- Line 599: Is Hellmanex III common knowledge? It seems like this may need a citation or source.

Added company and location to Materials and Methods for those interested in repeating our experiments. Hellmanex has been used in coverslip passivation for over 10 years.

Second decision letter

MS ID#: JOCES/2022/260621

MS TITLE: Cik1 and Vik1 Accessory Proteins Confer Distinct Functions to the Kinesin-14, Kar3

AUTHORS: Zane J Bergman, Jonathan J Wong, David Drubin, and Georjana Barnes
ARTICLE TYPE: Research Article

We have now reached a decision on the above manuscript.

© 2023. Published by The Company of Biologists under the terms of the Creative Commons Attribution License (https://creativecommons.org/licenses/by/4.0/).

To see the reviewers' reports and a copy of this decision letter, please go to: https://submit-jcs.biologists.org and click on the 'Manuscripts with Decisions' queue in the Author Area. (Corresponding author only has access to reviews.)

As you will see, the reviewers gave favourable reports but some critical points remained that will require amendments to your manuscript. I hope that you will be able to carry these out because I would like to be able to accept your paper, depending on further comments from reviewers.

Please ensure that you clearly highlight all changes made in the revised manuscript. Please avoid using 'Tracked changes' in Word files as these are lost in PDF conversion.

I should be grateful if you would also provide a point-by-point response detailing how you have dealt with the points raised by the reviewers in the 'Response to Reviewers' box. Please attend to all of the reviewers' comments. If you do not agree with any of their criticisms or suggestions please explain clearly why this is so.

Reviewer 1

Advance summary and potential significance to field

The revised manuscript effectively addresses my concerns.

Comments for the author

I have no further suggestions.

Reviewer 2

Advance summary and potential significance to field

This study uses an original in vitro assay with yeast cell lysate to show that the heterodimeric kinesins Kar3Vik1 and Kar3Cik1 have different effects on microtubule dynamics depending on the cell cycle state of the extract. The authors also report cell cycle dependent differences in some aspects of the microtubule plus end tracking behaviour of one of the heterodimers. This extends the knowledge of the behavior of these proteins beyond what was known from previous studies in cells and with purified proteins.

Comments for the author

The authors have not made an attempt to address the three rather minor concerns of this reviewer in the revised version of the manuscript (potential limitation of the assay, potential co-degradation, motivation and context for the performed experiments) although they claim to have done so in their replies to two of the concerns. It would be more straightforward just to explain why they felt that addressing these concerns in the manuscript was not necessary.

It would be useful to state whether the reported errors in Table 1 are SD or SEM or something else.

Reviewer 3

Advance summary and potential significance to field

I commend the authors for having done a significant amount of work to improve this manuscript. It will be a meaningful and impactful contribution to the literature. However, there are several issues that need to be addressed before it is suitable for publication.

© 2023. Published by The Company of Biologists under the terms of the Creative Commons Attribution License (https://creativecommons.org/licenses/by/4.0/).

Comments for the author

Significant issues:
1) This conclusion is not supported and must be adjusted accordingly. On Line 420 it is stated, “Interestingly, in both Cik1 and Vik1 depletions, the number of MTs with static, lateral interactions with GFP-Kar3 increased as compared to WT.” This is in reference to metaphase cell extracts. Critically, the percentage only increases from ~13 to ~17% percent. It is doubtful these differences would be significant if the error bars were SD, however they are SEM. With the current data there are no discernable differences between these percentages. Considering the SEM the p value will likely be close to 1. The conclusion should be that there is no observable difference.

2) The authors should take a very close look at the data presentation in the manuscript. While I did not check every number and/or graph, there are many that were noticed while reading the text and following the figures. For example:
Line 124 says 77.3% but Fig 1b appears to report 77.8%;
Line 126 states 96.5% and Fig 1b reports 95.4%;
Line 199 reports 31.6% whereas the corresponding values in Fig. 2C (16.1 + 14.5) are 30.6%;
Line 427: “In contrast, Vik1 depletion decreased the number of MTs with GFP-Kar3 on the minus-end from 18.4% in WT to 7.5%,” Figure 5E does not show 18.4% in WT extracts. The percentage shown for WT is clearly less than this.

3) The opening to the paragraph starting on Line 342 is still very troubling. It states, “Upon acute Kar3, Cik1, or Vik1 depletion via the AID degron system in lysates from metaphase-arrested cells, it became clear that Kar3Cik1 and Kar3Vik1 perform different functions in MT dynamics regulation.” This paragraph then goes on to describe how the effects of depleting either Vik1 and Cik1 essentially mirror each other, and then “These data suggest that, during metaphase, Kar3Cik1 and Kar3Vik1 regulate MT dynamics by similar mechanisms in vivo…” as well as depleting both may be synergistic in vivo. Critically, this data does not show that Kar3Cik1 and Kar3Vik1 perform different functions in MT dynamics regulation. On the contrary, it shows that they may perform similar functions in metaphase, only in different compartments. This needs to be corrected.

4) There are still issues citing and/or describing data. Line 375 states: “In total, these results mirror MT behavior observed in late anaphase cells, wherein cMTs and kinetochore MTs (kMTs) are kept short and stable by Kar3 heterodimers in their respective cellular compartments.” At no place in this manuscript is data presented showing that kMTs in late anaphase are kept short and stable by Kar3 heterodimers in their respective cellular compartments. There are also no citations to studies showing this. The authors may infer an effect on dynamic stability from the in vitro data, but this also carries the caveat that nuclear and cytoplasmic factors are mixed in this assay. Furthermore, not length or stability data is gathered for kMTs in vivo. This needs to be corrected and the manuscript should be carefully evaluated for similar issues.

5) Line 190: The text states “During metaphase in cells depleted of Cik1, GFP-Kar3 was absent from the nucleus, but a strong GFP-Kar3 signal was observed in the cytoplasm near the SPB in 98.7% of cells.” This claim is not supported by the data in Fig. 2A. In fact, there is a rather small Kar3 foci or two in the cells but the cloud of cloud of tubulin signal appears to encompass the Kar3 signal. It is very difficult, if at all, to tell the location of the SPB in these images. I would suggest the authors acknowledge this and/or clarifying this point using DAPI staining or an SPB marker.

6) Line 208: It appears the identical data measured for Fig. 1D are used in Fig. 2C (as a reference). Ideally these WT data should have auxin treatment like the other cells in Fig. 2C. The authors acknowledge the use of this data as “The WT values were the same as those determined in Fig. 1C.” However, this is somewhat unclear because it sounds very similar in meaning to the sentence “The WT values were similar as those determined in Fig. 1C.” I suggest the authors modify this sentence to read something more precise, for example “The WT values measured in Fig. 1C are presented for reference in Fig. 2D.”

7) Figure 5E and 5F are used to draw several conclusions, yet no statistical analyses appear to be reported. This seems like a critical missing element, especially considering the relative SEM compared to percentage changes in some conditions. Why are no statistics presented for Figure 5?

© 2023. Published by The Company of Biologists under the terms of the Creative Commons Attribution License (https://creativecommons.org/licenses/by/4.0/).

Minor comments:

- Just a suggestion that, since it is helpful to a reader refer to Fig. 1 when assessing the results in Fig. 2, it may be useful to present the images of Tubulin, Kar3, and merged in the same order in both figures.

- As the authors note, they previously showed that While WT lysates from asynchronous cells cannot assemble MTs, similar lysates from vik1∆ cells, but not from cik1Δ cells can assemble MTs. Thus, it is interesting, but not discussed or acknowledged, that cik1Δ cell lysates from all 4 of the cell cycle stages examined can assemble MTs. This seems interesting considering that the mixture of the 4 presumably cannot assemble MTs.

- Typo Line 602: "They seem to function seem by balancing"

Second revision

Author response to reviewers' comments

Reviewer 1 Advance Summary and Potential Significance to Field:
The revised manuscript effectively addresses my concerns.

Reviewer 1 Comments for the Author:
I have no further suggestions.

Reviewer 2 Advance Summary and Potential Significance to Field:
This study uses an original in vitro assay with yeast cell lysate to show that the heterodimeric kinesins Kar3Vik1 and Kar3Cik1 have different effects on microtubule dynamics depending on the cell cycle state of the extract. The authors also report cell cycle dependent differences in some aspects of the microtubule plus end tracking behaviour of one of the heterodimers. This extends the knowledge of the behavior of these proteins beyond what was known from previous studies in cells and with purified proteins.

Reviewer 2 Comments for the Author:
The authors have not made an attempt to address the three rather minor concerns of this reviewer in the revised version of the manuscript (potential limitation of the assay, potential co-degradation, motivation and context for the performed experiments) although they claim to have done so in their replies to two of the concerns. It would be more straightforward just to explain why they felt that addressing these concerns in the manuscript was not necessary. It would be useful to state whether the reported errors in Table 1 are SD or SEM or something else.

Though we addressed caveats and shortcomings of our assay in our previous version of the manuscript, we have added these in our Discussion (see paragraph starting on line 510). We hope this revision better calls the reader's attention to these issues.
We have performed a Western blot analysis of strains arrested at metaphase and depleted of Cik1 or Vik1 and probed for GFP-Kar3 levels, now included in the supplemental figures (Fig. S2B). The amount of Kar3 does not decrease when either accessory protein is depleted.
Upon re-reading the manuscript, the authors believe that the motivations for the experiments have been sufficiently explained.
We have also amended the text to indicate that the errors reported in Table 1 are SD and those in Table 2 are SEM.

Reviewer 3 Advance Summary and Potential Significance to Field:
I commend the authors for having done a significant amount of work to improve this manuscript. It will be a meaningful and impactful contribution to the literature. However, there are several issues that need to be addressed before it is suitable for publication.

Reviewer 3 Comments for the Author:
Significant issues:

© 2023. Published by The Company of Biologists under the terms of the Creative Commons Attribution License (https://creativecommons.org/licenses/by/4.0/).

1) This conclusion is not supported and must be adjusted accordingly. On Line 420 it is stated, “Interestingly, in both Cik1 and Vik1 depletions, the number of MTs with static, lateral interactions with GFP-Kar3 increased as compared to WT.” This is in reference to metaphase cell extracts. Critically, the percentage only increases from ~13 to ~17% percent. It is doubtful these differences would be significant if the error bars were SD, however they are SEM. With the current data there are no discernable differences between these percentages. Considering the SEM the p value will likely be close to 1. The conclusion should be that there is no observable difference.

The reviewer is correct in that the difference observed between conditions is not sufficiently pronounced enough to be able to draw any strong conclusions. The closing statement has been omitted.

2) The authors should take a very close look at the data presentation in the manuscript. While I did not check every number and/or graph, there are many that were noticed while reading the text and following the figures. For example: Line 124 says 77.3% but Fig 1b appears to report 77.8%;
Line 126 states 96.5% and Fig 1b reports 95.4%;
Line 199 reports 31.6% whereas the corresponding values in Fig. 2C (16.1 + 14.5) are 30.6%;
Line 427: “In contrast, Vik1 depletion decreased the number of MTs with GFP-Kar3 on the minus-end from 18.4% in WT to 7.5%,” Figure 5E does not show 18.4% in WT extracts. The percentage shown for WT is clearly less than this.

This is a helpful suggestion and Fig. 2 has been changed to mirror the order of Fig. 1.

- As the authors note, they previously showed that While WT lysates from asynchronous cells cannot assemble MTs, similar lysates from vik1Δ cells, but not from cik1Δ cells can assemble MTs. Thus, it is interesting, but not discussed or acknowledged, that cik1Δ cell lysates from all 4 of the cell cycle stages examined can assemble MTs. This seems interesting considering that the mixture of the 4 presumably cannot assemble MTs.

A couple of key differences are at play here:
1) We are not using cik1Δ strains, instead we use CIK1-AID. The AID system was used to get around chronic MT aberrations in the deletion background.
2) We do not get MT assembly in lysates from asynchronous WT cells, so the vik1Δ is the outlier.
3) Our previous paper showed that mixing G1-arrested lysate with S phase-arrested lysate from WT cells would prevent MT assembly. We did not try mixing other combinations of lysates. It seems that the mysterious G1 factor is dominant with respect to licensing MT assembly in asynchronous lysates. The interesting result is that MTs assemble in G1-arrested lysates with either Kar3, Cik1, or Vik1 depleted, which we do discuss.

- Typo Line 602: “They seem to function seem by balancing”

Thank you for pointing out this error. It has been corrected.

Third decision letter

MS ID#: JOCES/2022/260621

MS TITLE: Cik1 and Vik1 Accessory Proteins Confer Distinct Functions to the Kinesin-14, Kar3

AUTHORS: Zane J Bergman, Jonathan J Wong, David Drubin, and Georjana Barnes
ARTICLE TYPE: Research Article

I am happy to tell you that your manuscript has been accepted for publication in Journal of Cell Science, pending standard ethics checks.

© 2023. Published by The Company of Biologists under the terms of the Creative Commons Attribution License (https://creativecommons.org/licenses/by/4.0/).
